# A Novel Sulfatase for Acesulfame Degradation in Wastewater Treatment Plants as Evidenced from *Shinella* Strains

**DOI:** 10.1021/acs.est.4c02283

**Published:** 2024-10-07

**Authors:** Yu Liu, Thore Rohwerder, Maria L. Bonatelli, Theda von Postel, Sabine Kleinsteuber, Lorenz Adrian, Chang Ding

**Affiliations:** † Molecular Environmental Biotechnology, Helmholtz Centre for Environmental Research − UFZ, Leipzig 04318, Germany; ‡ Chair of Geobiotechnology, Technische Universität Berlin, 13355 Berlin, Germany; § Microbial Biotechnology, Helmholtz Centre for Environmental Research − UFZ, Leipzig 04318, Germany

**Keywords:** Sequence Read Archive, metagenome-assembled genomes, xenobiotic compounds, sewage, global distribution, pathway evolution, sulfuric ester hydrolase

## Abstract

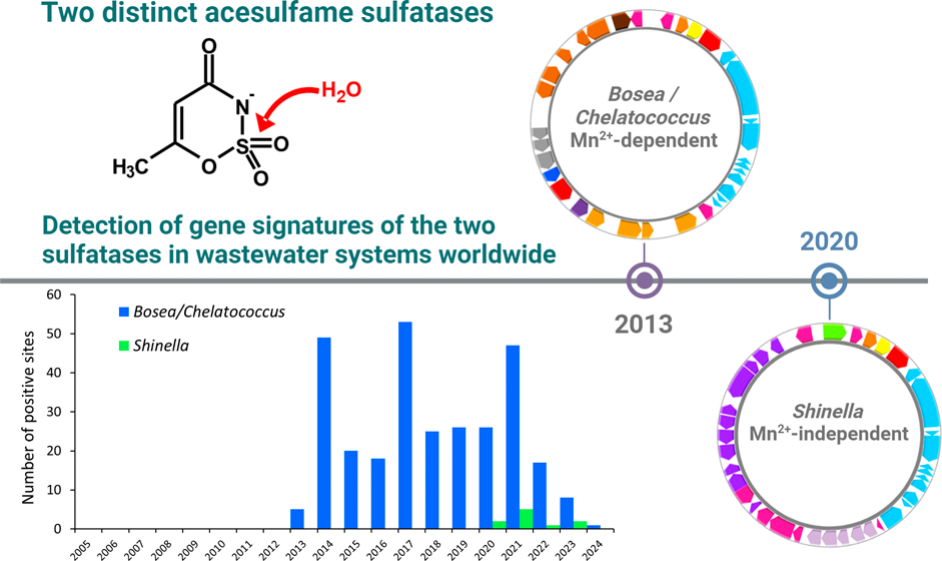

The artificial sweetener acesulfame is a persistent pollutant in wastewater worldwide. So far, only a few bacterial isolates were recently found to degrade acesulfame efficiently. In *Bosea* and *Chelatococcus* strains, a Mn^2+^-dependent metallo-β-lactamase-type sulfatase and an amidase signature family enzyme catalyze acesulfame hydrolysis via acetoacetamide-*N*-sulfonate to acetoacetate. Here, we describe a new acesulfame sulfatase in *Shinella* strains isolated from wastewater treatment plants in Germany. Their genomes do not encode the Mn^2+^-dependent sulfatase. Instead, a formylglycine-dependent sulfatase gene was found, together with the acetoacetamide-*N*-sulfonate amidase gene on a plasmid shared by all known acesulfame-degrading *Shinella* strains. Heterologous expression, proteomics, and size exclusion chromatography corroborated the physiological function of the *Shinella* sulfatase in acesulfame hydrolysis. Since both acesulfame sulfatase types are absent in other bacterial genomes or metagenome-assembled genomes, we surveyed 73 tera base pairs of wastewater-associated metagenome raw data sets. *Bosea*/*Chelatococcus* sulfatase gene signatures were regularly found from 2013, particularly in North America, Europe, and East Asia, whereas *Shinella* sulfatase gene signatures were first detected in 2020. Moreover, signatures for the *Shinella* sulfatase and amidase genes co-occur only in six data sets from China, Finland, and Mexico, suggesting that the *Shinella* genes were enriched or introduced quite recently in wastewater treatment facilities.

## Abstract 2

A novel sulfatase that hydrolyzes the once recalcitrant xenobiotic acesulfame was identified. Surveying metagenome data sets revealed a very recent enrichment or introduction of its gene in wastewater treatment systems worldwide.

## Introduction

Acesulfame is one of the most commonly used artificial sweeteners in low-calorie food and beverages, pharmaceuticals and cosmetics.^1,2^ The global consumption of acesulfame, which is excreted unchanged via the kidney,^2^ led to its widespread presence in domestic wastewater (typically between 10 and 100 μg L^–1^),^3^ groundwater (up to 5 μg L^–1^),^4^ seawater (0.57–9.9 μg L^–1^),^5^ and tap water (up to 2.6 μg L^–1^).^6^ Acesulfame concentrations of up to 2.5 mg L^–1^ were reported in effluents from European wastewater treatment plants (WWTPs).^7^ Even though the approved usage of acesulfame as a food additive indicates its low estimated risks for human health and the environment, several studies highlighted potential risks associated with acesulfame and its transformation products. One example is that acesulfame acts as an inhibitor of P-glycoprotein, thereby reducing the detoxification capacity of the liver.^8^ In addition, it also induces uterine hypercontraction upon long-term high-dose exposure^9^ and might cause DNA damage^10^ through the formation of complexes in the minor groove of DNA.^11^ Recent studies reported oxidative stress in fish^12^ and neurotoxic effects in *Daphnia magna*(13) upon acesulfame exposure. Moreover, acesulfame can be transformed into more persistent and more toxic products by chlorination,^14^ UV irradiation,^15^ and TiO_2_-assisted photolysis,^16,17^ posing a long-term potential risk for aquatic environments. For example, the toxicity of acesulfame transformation products after UV/TiO_2_ treatment was found to be magnified by a factor of 575 compared to that of acesulfame itself.^16^ Additionally, it has been observed that acesulfame can promote the horizontal dissemination of antibiotic resistance genes in pure culture systems,^18^ in gut microbiome,^19^ and during anaerobic digestion.^20^

Previously, acesulfame was regarded as an ideal tracer to identify anthropogenic contamination sources^6,21^ due to its persistence and mobility in aquatic systems.^22^ However, recent studies showed that acesulfame can actually be biodegraded,^23,24^ which makes its use as conservative tracer obsolete. Nevertheless, since biodegradation is temperature-dependent,^24,25^ acesulfame can still be used as a transient tracer in cold season (<10 °C).^26,27^

**Figure 1:**
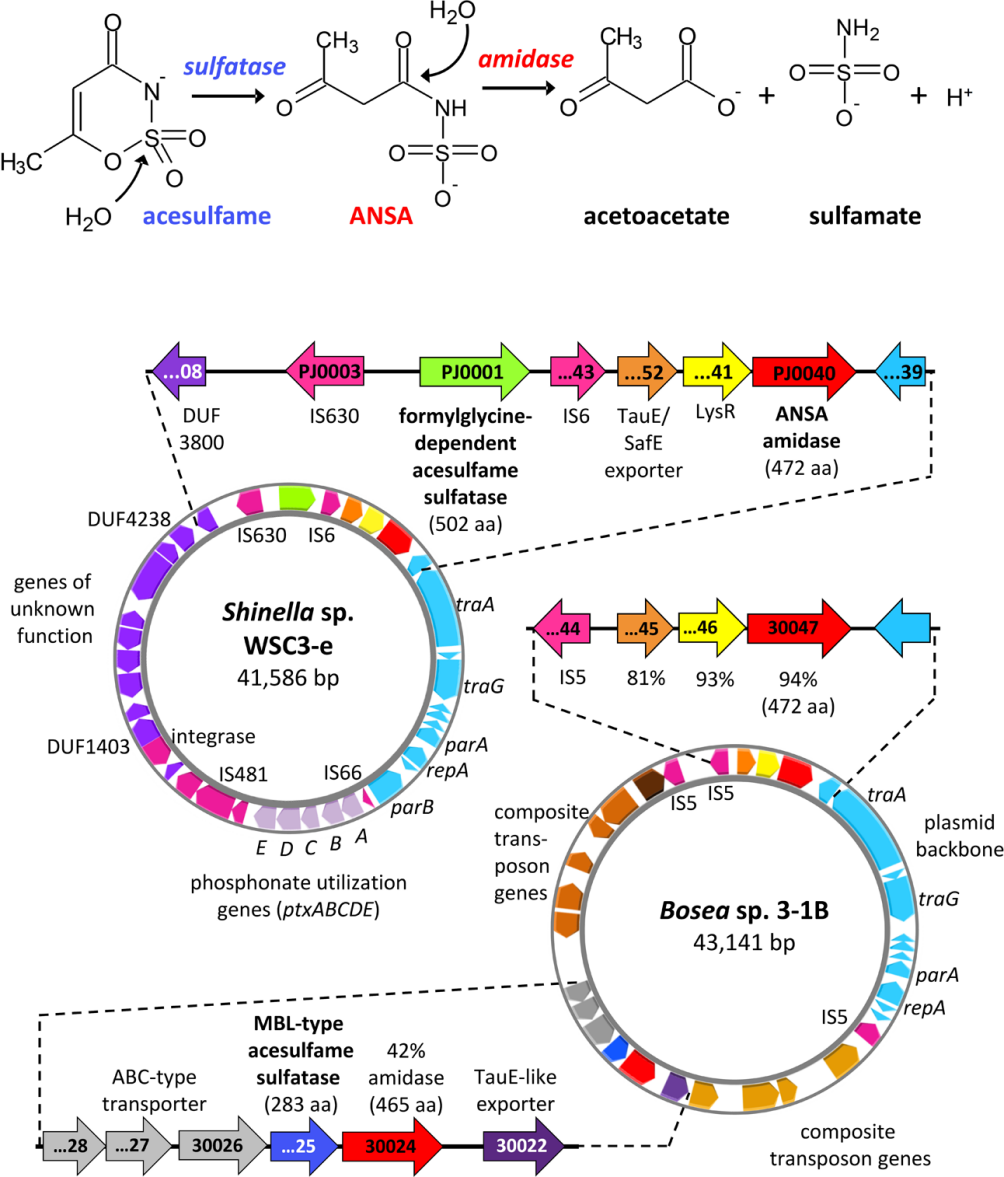
Two-step enzymatic hydrolysis of acesulfame (upper panel) and comparison of AUP plasmids between acesulfame-degrading *Shinella* and *Bosea*/*Chelatococcus* strains (lower panel). In *Bosea* sp. 3-1B, an MBL-type acesulfame sulfatase is encoded in a six coding sequence spanning metabolic gene cluster embedded in a composite transposon, while the ANSA amidase gene is located in another gene cluster together with two coding sequences encoding a LysR-type regulator and a TauE/SafE export system. This ANSA amidase cluster is fully conserved in *Shinella* sp. WSC3-e. However, the MBL-type acesulfame sulfatase is replaced by a formylglycine-dependent acesulfame sulfatase, which is not encoded in a separate gene cluster but directly upstream of the ANSA amidase cluster. Labels within the gene symbols refer to locus tags (prefix BOSEA31B_ and SHIWSC3_ for strains 3-1B and WSC3-e, respectively). The amino acid sequence identities (blastp) of *Bosea* ANSA amidase, LysR, and TauE/SafE proteins to the respective *Shinella* sequences are indicated. Additionally, the identity (blastp) of the second *Bosea* AUP plasmid amidase (BOSEA31B_30024) to the *Shinella* ANSA amidase is given. Both AUP plasmids are shown true to scale. Annotation details for the *Shinella* AUP plasmid are given in Table S5. The plasmid figure was generated with Proksee.

Thus far, 14 bacterial strains able to grow with acesulfame as sole carbon and energy source have been isolated from wastewater habitats. They belong to the genera *Bosea* (strains 3-1B and 100-5),^28^*Chelatococcus* (strains 1g-2, 1g-11, WSA4-1, WSC3-1, WSD1-1, WSG2-a, YT9, and HY11),^28−30^ and *Shinella* (strains HY16, YE25, YZ44, and WSC3-e).^30,31^ In addition, we recently isolated *Shinella* sp. WSD5-1, which is also able to grow on acesulfame. *Bosea* sp. 3-1B, which was obtained from a treatment wetland in Germany sampled in 2015, was the first acesulfame-degrading bacterial isolate worldwide.^28^ In *Bosea* and *Chelatococcus* strains, acesulfame is hydrolyzed via acetoacetamide-N-sulfonate (ANSA) to acetoacetate and sulfamate. In this pathway, a Mn^2+^-dependent metallo-β-lactamase (MBL)-type sulfatase catalyzes the nucleophilic attack of acesulfame by a water molecule to form ANSA (Figure 1).^30^ Subsequently, the amide bond of ANSA is cleaved by an amidase signature family enzyme.^30^ However, in the genomes of the above-mentioned *Shinella* strains^30,31^ and also in our newly sequenced strain *Shinella* sp. WSD5-1, the gene for the Mn^2+^-dependent MBL-type sulfatase is missing (as confirmed by using tblastn), while the ANSA amidase gene known from the *Bosea*/*Chelatococcus* genomes is conserved.

Based on these findings, we hypothesized that in *Shinella* strains a different hydrolase may be responsible for the acesulfame hydrolysis step, and therefore aimed to identify this novel acesulfame-hydrolyzing enzyme in *Shinella* sp. WSC3-e. Through heterologous expression of a candidate gene, activity assays and proteomic analysis, we discovered a novel formylglycine-dependent sulfatase and experimentally confirmed its acesulfame-hydrolyzing activity. To obtain indications about the historic occurrence and geographic distribution of the acesulfame degradation gene sequences, we analyzed wastewater-derived metagenome raw sequence data sets for the occurrence of both *Shinella* and *Bosea*/*Chelatococcus* acesulfame sulfatase and ANSA amidase gene signatures.

## Materials and Methods

### Cultivation of *Shinella* Strains and Preparation of Crude Extracts

*Shinella* sp. WSC3-e was previously isolated from activated sludge of the WWTP Rosental Leipzig, Germany.^30^*Shinella* sp. WSD5-1 was isolated as described for strain WSC3-e^30^ from the WWTP of Markkleeberg, Germany, which was sampled in 2020. Both strains showed the capability of growing on acesulfame as the sole carbon and energy source. To study growth and degradation kinetics, strainWSC3-e was cultivated in mineral medium (DSMZ 462, containing 0.15 μM MnCl_2_) with 30 mM acesulfame at 125 rpm and 30 °C. A Mn^2+^-rich control was incubated in the same medium supplemented with 3.7 μM MnCl_2_. Protein crude extracts of *Shinella* sp. WSC3-e were obtained from late logarithmic cells (30 °C, 125 rpm, DSMZ 462 medium with 30 mM acesulfame as sole source of carbon and energy) that were harvested by centrifugation at 6000*g* and 4 °C and disrupted in lysis buffer (50 mM Tris-HCl with 2 mM dithiothreitol, DTT, 5 mM MgCl_2_ and 10% glycerol, pH 7.8) in a mixer mill with glass beads.^30^ Crude extracts were obtained by taking supernatant after centrifugation at 20,000*g* and 4 °C for 20 min.

### Genome of Strain WSD5-1 and Comparison of *Shinella* Genomes

The genome of strain WSD5-1 was sequenced by combining short- and long-read techniques as described in Text S1 and deposited at the European Nucleotide Archive under the project PRJEB50809. The FastANI software^32^ in the Proksee system^33^ was used to calculate the pairwise average nucleotide identity of the *Shinella* sp. WSC3-e genome (GenBank accession number GCA_945994535.2) with the genomes of strains *Shinella* sp. WSD5-1 (GCA_963942435.1), YE25 (GCA_028534295.1), and HY16 (GCA_028534175.1). Query coverage values based on the number of orthologous fragments.

### Heterologous Expression of the Sulfatase Gene SHIWSC3_PJ0001 in *Escherichia coli*

SHIWSC3_PJ0001 was synthesized and cloned (via *Nde*I/*Bam*HI sites) without the initial 36 bp by GenScript (Oxford, United Kingdom) into the expression vector pET-28a(+)-TEV. The recombinant plasmid was transformed into *E. coli* Lemo21 (DE3) (New England Biolabs), which was grown at 30 °C and 150 rpm in lysogeny broth with 30 mg L^–1^ chloramphenicol and 50 mg L^–1^ kanamycin until an optical density (600 nm) of 0.5 was reached. Then, 0.4 mM of isopropyl β-d-1-thiogalactopyranoside was added and incubated further at 30 °C and 150 rpm for 4 h. Cells were harvested by centrifugation at 6,000*g* and 4 °C. As a control experiment, a gene encoding a 465-aa amidase signature enzyme from *Bosea* sp. 100-5 (BOSEA1005_40016), which is not involved in acesulfame or ANSA degradation,^30^ was expressed from pET-28a (+)-TEV under the same conditions.

### Acesulfame-Hydrolyzing Activity in *E. coli* Crude Extracts

Harvested *E. coli* cells were disrupted as described above for the *Shinella* cells. The *E. coli* crude extracts were tested for acesulfame-hydrolyzing activity. The activity assay contained 50 mM Tris-HCl (pH 7.8), 10% glycerol, 2.25 mM acesulfame (potassium salt, 99% purity, Merck, Darmstadt, Germany), 2 mM adenosine 5′-triphosphate (ATP), 2 mM DTT, 5 mM MgCl_2_, and crude extract (added in the end to start the reaction). Incubation was done at 30 °C and 300 rpm. Reactions were stopped by adding two volumes of 10 mM sodium malonate buffer (pH 4.0, preheated to 60 °C) to assay samples and incubating the mixture at 60 °C for 5 min. Then, acesulfame and ANSA concentrations were quantified as described in section Analytical methods.

### Size Exclusion Chromatography (SEC) and Shotgun Proteomics

To obtain protein fractions from SEC with acesulfame-hydrolyzing activity, 200 μL crude extract from *Shinella* sp. WSC3-e cells (1.6 μg μL^–1^ protein) was loaded onto a Superdex 200 10/300 column (GE Healthcare, Chicago, USA), which was equilibrated with a buffer containing 50 mM Tris-HCl (pH 7.84), 10% glycerol, 2 mM ATP, 2 mM DTT and 5 mM MgCl_2_ at 0.5 mL min^–1^. Fractions of 0.5 mL between elution volumes 7 and 22 mL were collected, which were used for both acesulfame-hydrolyzing activity assay and proteomic analysis. The activity assay contained 78 μL SEC fraction subsample and 2 μL of 90 mM acesulfame stock solution (final concentration: 2.25 mM) and was incubated at 30 °C and 400 rpm for 40 min before being stopped in preheated malonate buffer as described for the *E. coli* extracts. Hydrolase activity was calculated from end-point acesulfame concentrations. For assigning SEC retention times to molecular masses, the system was calibrated using the Protein Standard Mix 15–600 kDa (Sigma-Aldrich, Darmstadt, Germany). Calibration was done under a flow rate of 0.5 mL min^–1^ with a mobile phase containing 50 mM Tris-HCl buffer (pH 7.5), 10% glycerol, and 0.03% (w/v) digitonin. The shotgun proteomics of SEC fractions and crude extract was measured after tryptic digestion on a nano-LC-MS/MS (Orbitrap) as described in Text S2. The proteomics data were deposited at ProteomeXchange via the PRIDE partner repository with the number PXD046105.

### Analytical Methods

Protein concentration was determined with the bicinchoninic acid method, using the Pierce BCA Protein Assay Kit (Thermo Fisher Scientific, Massachusetts, USA) according to the manufacturer’s instruction, or Bradford reagent (AppliChem, Darmstadt, Germany), using bovine serum albumin as standard. Acesulfame and ANSA were quantified using HPLC and LC-MS/MS, respectively, as previously described.^30^

### Database Search

The blastp and blastn tools were used to search for sequences related to the acesulfame sulfatase (SHIWSC3_PJ0001) and ANSA amidase (SHIWSC3_PJ0040) from *Shinella* sp. WSC3-e in nonredundant sequence database and whole genome shotgun contigs databases, respectively (Text S3). Furthermore, raw sequence files of shotgun metagenome and metatranscriptome projects in the context of wastewater environments ((Table S1) and human gut microbiome (Table S2) were downloaded from the NCBI Sequence Read Archive (SRA) and searched with blastn using the acesulfame sulfatase and ANSA amidase genes from *Bosea*/*Chelatococcus*^30^ and *Shinella* as query (Text S4 and Text S5). In the SRA search, a significant match was defined as the alignment of single ≥150-bp reads or the combination of shorter sequences (e.g., two 100-bp reads) resulting in ≥140 bp query coverage at ≥97% identity (query coverage is shown in Table S3). SRA reads having incomplete alignment due to matching only with gene start or end regions (≥30 bp at 97% identity) were also checked for possible alignments with sequences flanking the corresponding sulfatase and amidase genes in the respective genomes (*Shinella* sp. WSC3-e and *Bosea* sp. 100-5). If those reads matched the flanking sequences and fulfilled the alignment criteria above, they were also considered significant matches. These SRA search criteria are highly specific for the four query genes, as tested with the SRA data sets of human gut microbiome (Text S5). Moreover, the criteria allow clearly distinguishing between the *Shinella* query genes and their closest relatives (Text S6 and Text S7). A sampling site containing reads of significant matches to a query gene at a specific year was counted as one positive sampling site of this query gene, regardless how many reads were associated with the detection.

## Results

### Genomes of Acesulfame-Degrading *Shinella* Strains

The previously sequenced genomes of acesulfame-degrading *Shinella* strains YE25, HY16^31^ and WSC3-e^30^ as well as the genome of the new isolate *Shinella* sp. WSD5-1 were compared. Although isolated from different locations, strains YE25 (WWTP Sha Tin, Hong Kong, China), WSD5-1 (WWTP Markkleeberg, Germany) and WSC3-e (WWTP Rosental, Leipzig, Germany) possess almost identical genomes (≥99% average nucleotide identity at >99% coverage when pairwise compared with WSC3-e). Moreover, replicon organization is similar in these strains, representing a chromosome of 4.7 mega base pairs (Mbp) and up to 12 plasmids (Table S4, Figure S1). In two cases, two smaller plasmids of strains YE25 and WSD5-1 were rearranged to larger ones in strain WSC3-e (Figure S1). All three genomes hold a total sequence length of 7.8 Mbp. In contrast, the genome of strain HY16 (also from WWTP Sha Tin) only amounts to 7.3 Mbp and presents only 90.8% average nucleotide identity at 74% query coverage with *Shinella* sp. WSC3-e.

### One of the Plasmids Harbors Genes for Acesulfame Degradation

A closer inspection of the genomes revealed that the four *Shinella* strains have an almost identical 41.6 kilo base-pair (kbp) plasmid (plasmid PJ in strain WSC3-e, OZ000540) with 100% conservation of all coding sequences (termed *Shinella*acesulfame utilization pathway, AUP, plasmid) (Figure 1). The *Shinella* AUP plasmid harbors the ANSA amidase gene cluster encoding the second step of the acesulfame degradation pathway previously described for *Bosea* and *Chelatococcus* strains.^30^ Among all the AUP-bearing strains, the ANSA amidase gene cluster shows variation in length, ranging from two coding sequences in *Bosea* sp. 100-5 to four in *Chelatococcus* sp. WSC3-1 and 1g-11.^30^ The *Shinella* ANSA amidase gene cluster shows the highest similarity with the three coding sequences-bearing cluster found in strain *Bosea* sp. 3-1B (Figure 1). Besides the gene for the ANSA amidase (SHIWSC3_PJ0040), two other coding sequences (SHIWSC3_PJ0052 and _PJ0041) are present in the *Shinella* ANSA amidase gene cluster, encoding a TauE/SafE export protein and a LysR-like transcriptional regulator, respectively. In addition to the ANSA amidase gene cluster, the *Shinella* AUP plasmid encodes several backbone genes involved in conjugative transfer, replication and maintenance, which are homologous to and syntenic with their counterparts on the *Bosea* AUP plasmid (Figure 1), e.g., the transfer and replication genes *traA*, *traG* and *parA*. This is indicative of a common origin of the plasmids.

On the other hand, the *Shinella* AUP plasmid harbors a gene cluster annotated to encode proteins involved in phosphonate utilization, several insertion sequence (IS) elements and other genes that are not present on the *Bosea* and *Chelatococcus* AUP plasmids (Figure 1, Table S5). One of these genes (SHIWSC3_PJ0001) is located directly upstream of the ANSA amidase gene cluster and encodes a protein annotated as formylglycine-dependent sulfatase with a length of 502 aa. Although the sulfatase motif (CxPxRxxxLTGR) essential for the posttranslational modification of an active site cysteine residue (C64 in the *Shinella* sulfatase) into the catalytically active formylglycine residue^34^ is 100% conserved, the enzyme is only distantly related to biochemically characterized formylglycine-dependent sulfatases. For example, the *Shinella* sulfatase has 30% amino acid identity along 93% of the query sequence with the choline sulfatase from *Ensifer meliloti* (O69787). Therefore, its function and substrate specificity cannot be clearly assigned by sequence comparison. However, considering the absence of the *Bosea*/*Chelatococcus* MBL-type acesulfame sulfatase gene cluster in the *Shinella* strains^30^ and the necessity of a sulfatase to catalyze acesulfame degradation, we hypothesized that the SHIWSC3_PJ0001 gene product encodes a novel acesulfame sulfatase. The conversion of its active site cysteine residue could be catalyzed by two oxygen-dependent formylglycine-generating enzymes encoded in the WSC3-e genome by chromosomal SHIWSC3_1933 and SHIWSC3_PF0003 from plasmid PF (OZ000536) (see Text S8).

### Mn^2+^-Independent Hydrolysis of Acesulfame in *Shinella* sp. WSC3-e

**Figure 2:**
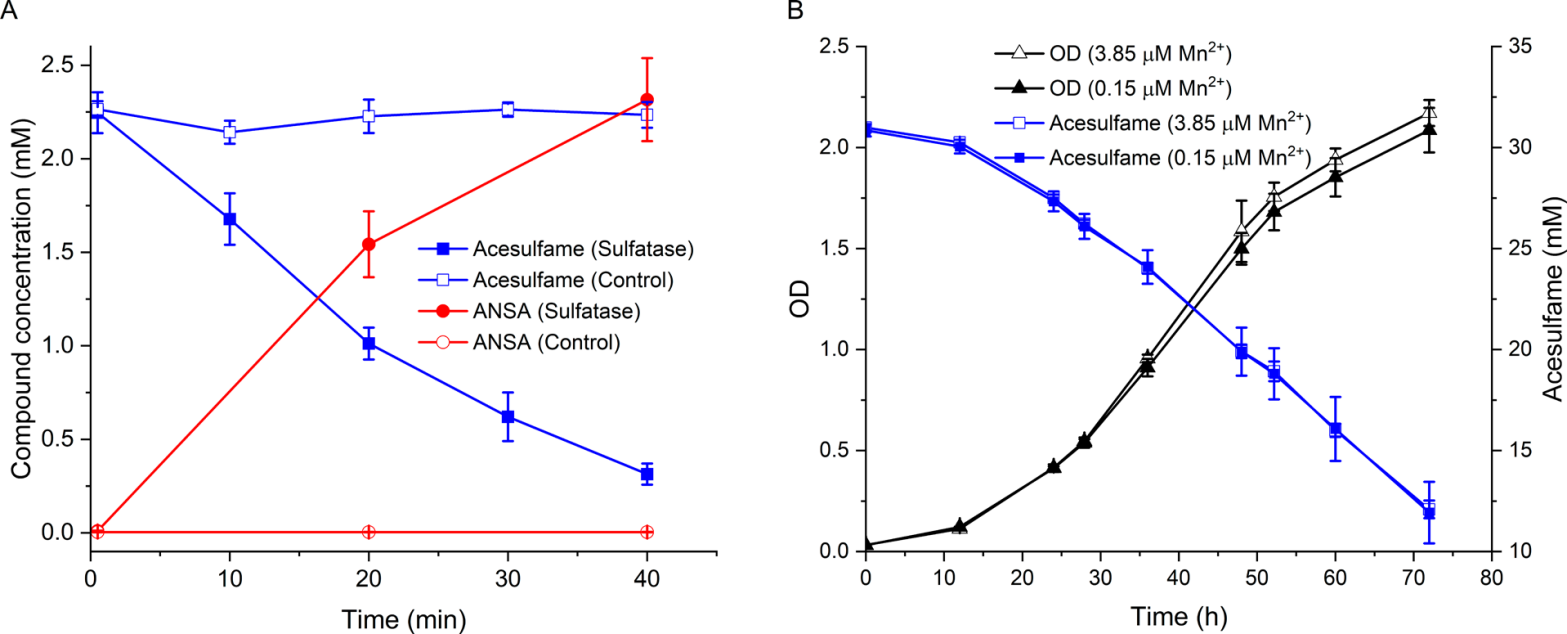
Biochemical and physiological characteristics of acesulfame degradation in *Shinella* sp. WSC3-e. (A) Stoichiometric conversion of acesulfame to ANSA by crude extracts from *E. coli* Lemo21 (DE3) cells expressing the *Shinella* sp. WSC3-e sulfatase gene SHIWSC3_PJ0001 (assay with 0.58 μg μL^–1^ total protein). The corresponding crude extract of an *E. coli* Lemo21 (DE3) strain without the SHIWSC3_PJ0001 gene was used as a control (0.57 μg μL^–1^ total protein) and showed neither acesulfame removal nor ANSA formation (detection limit: 0.2 μM with LC-MS/MS). (B) Incubation of *Shinella* sp. WSC3-e on acesulfame at 30 °C in Mn^2+^-poor (0.15 μM Mn^2+^) or Mn^2+^-rich mineral salt medium (3.85 μM) enabled growth with a doubling time of about 7 h. OD, optical density at 600 nm. Values given represent mean and standard deviation from five biological replicates.

For heterologous expression, we cloned the sulfatase gene SHIWSC3_PJ0001 into *E. coli* Lemo21 (DE3). In line with our hypothesis that the *Shinella* gene encodes an acesulfame sulfatase, crude extracts of the recombinant *E. coli* strain stoichiometrically converted acesulfame to ANSA, while a negative control obtained from cells incubated under the same conditions without the *Shinella* sulfatase gene did not (Figure 2A). This result also indicates that, in contrast to the *Bosea*/*Chelatococcus* MBL-type acesulfame sulfatase, the SHIWSC3_PJ0001-encoded enzyme is Mn^2+^-independent, as the lysogeny broth medium used for the heterologous expression in *E. coli* contains insufficient amounts of Mn^2+^ to enable appropriate loading of heterologous Mn^2+^-dependent proteins with this metal ion.^35^ Such Mn^2+^-independence of the *Shinella* acesulfame sulfatase was also corroborated by growing *Shinella* sp. WSC3-e on acesulfame as the sole carbon and energy source in a mineral salt medium that was either poor (0.15 μM) or rich (3.85 μM) in Mn^2+^, resulting in almost identical acesulfame transformation activity and growth (Figure 2B). In comparison, it was previously demonstrated that heterologous expression of the MBL-type acesulfame sulfatase in *E. coli* depends on supplementation of the lysogeny broth with 0.5 mM Mn^2+^ and that only the Mn^2+^-rich but not the Mn^2+^-poor mineral salt medium supported efficient growth and acesulfame degradation of *Bosea* sp. 100-5.^30^

### Proteome of *Shinella* sp. WSC3-e

Proteomic analysis of the crude extract (three replicates) of the acesulfame-grown *Shinella* sp. WSC3-e identified 17,839 peptides grouping into 2,498 proteins, out of 8,823 proteins predicted from the genome. The *Shinella* AUP plasmid-encoded SHIWSC3_PJ0001 sulfatase and the SHIWSC3_PJ0040 amidase were found among the 20 most abundant proteins (Table S6, Table S7), accounting for 6.7% and 1.2% of the proteome, respectively. Besides, several chromosomal genes encoding ABC-type transporter proteins and major chaperons (GroEL/GroES, DnaK systems) as well as a plasmid-borne (plasmid PD, OZ000534) gene encoding a chaperon ClpB-related protein were also strongly expressed. In regard to basic cellular functions, chromosome-encoded elongation factors and enzymes for the tricarboxylic acid cycle, for ammonia assimilation and for the glyoxylate cycle were most prominent. Additionally, several genes from plasmid PF were highly expressed (Table S6, Table S7). These encode an acetyl-CoA C-acetyltransferase (SHIWSC3_PF0360 gene product) and a couple of putative enzymes with less reliable functional annotation.

**Figure 3:**
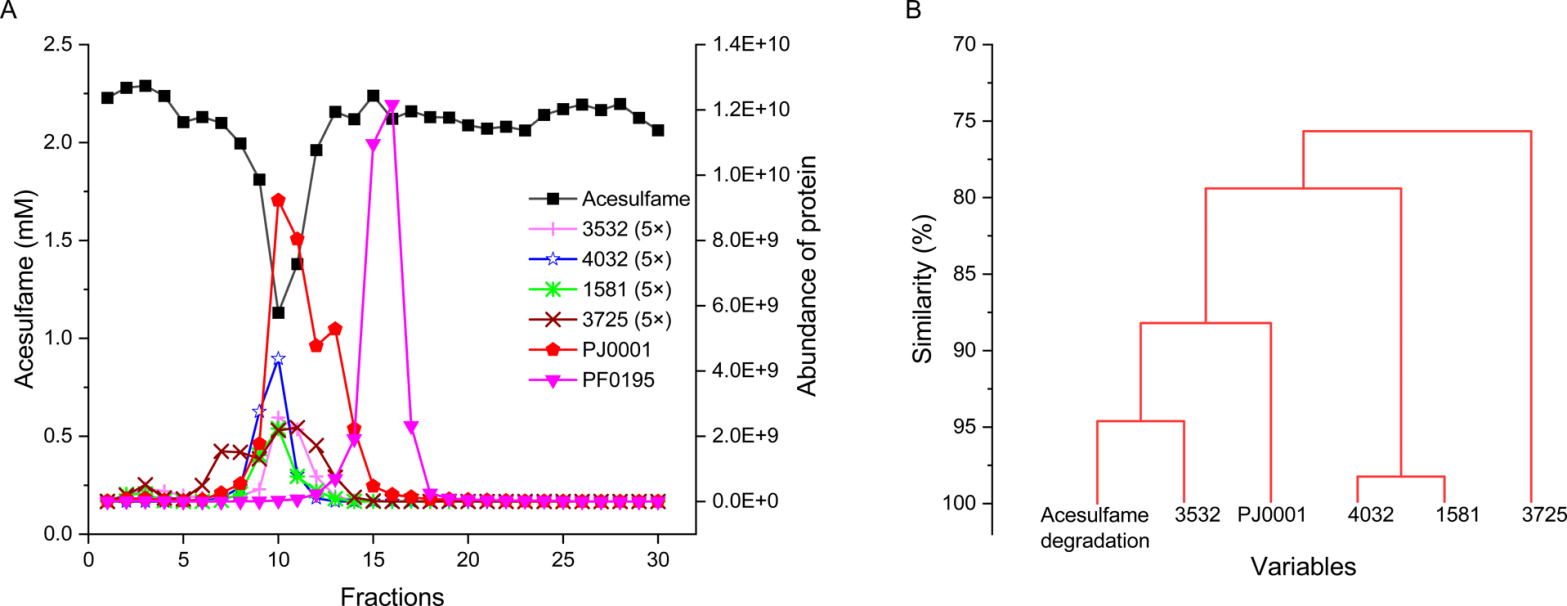
Fractionation of *Shinella* sp. strain WSC3-e crude extract by SEC and correlation of protein abundance with acesulfame sulfatase activity in the fractions. (A) SEC elution profiles of acesulfame degradation (remaining acesulfame concentration after incubating a subsample of each fraction with 2.25 mM acesulfame for 40 min) and abundance of the SHIWSC3_PJ0001 sulfatase, several coeluting proteins, and the SHIWSC3_PF0195 CocE/NonD family hydrolase. For better visibility, abundance values of proteins that coelute with acesulfame degradation activity were 5-fold increased (labeled with 5×), except SHIWSC3_PJ0001. (B) Subdendrogram of the hierarchical cluster analysis for acesulfame hydrolysis and abundance distribution of the 200 most abundant proteins found in the SEC fractions. Only the five proteins with a similarity value >75% are shown in this subdendrogram. Locus tags (prefix is SHIWSC3_) and their annotations: 3532, 2-oxoglutarate dehydrogenase; 4032, glutamate dehydrogenase; 1581, transcriptional repressor; 3725, pyruvate carboxylase.

Protein abundance and acesulfame sulfatase activity were analyzed in SEC fractions obtained from crude extracts of acesulfame-grown *Shinella* sp. WSC3-e. Acesulfame degradation activity peaked in fraction F10 (Figure 3A), corresponding to an apparent molecular mass of about 200 kDa. The SHIWSC3_PJ0001 sulfatase had its highest abundance in fraction F10 too, indicating its presence as a homotrimeric (171 kDa) or homotetrameric (228 kDa) form, based on a predicted monomeric size of 57 kDa. In addition, several other proteins with abundance lower than the sulfatase coeluted with acesulfame degradation activity (Figure 3A). In a hierarchical cluster analysis, the acesulfame-hydrolyzing activity was analyzed together with the abundance values of the 200 most abundant proteins found across the SEC fractions (Figure S2, Table S8). Five proteins showed a strong correlation between their abundance distribution and the distribution of acesulfame sulfatase activity across the SEC fractions (Figure 3B). Among these five proteins, the SHIWSC3_PJ0001 sulfatase was the only hydrolase according to the annotation. The activation of the SHIWSC3_PJ0001 sulfatase is possibly catalyzed by the SHIWSC3_PF0003 formylglycine-generating enzyme present in the proteome, as the SHIWSC3_1933-encoded enzyme was not detected (Table S7).

### Acesulfame Sulfatase and ANSA Amidase Genes in Public Sequence Databases

As outlined above, the *Shinella* AUP plasmid bearing the Mn^2+^-independent acesulfame sulfatase and the ANSA amidase genes is 100% conserved in all acesulfame-degrading *Shinella* strains isolated thus far. Certain features of the plasmid were also found to be 100% conserved in other bacteria, e.g., the IS481 family element together with the phosphonate utilization gene cluster in the genomes of *Agrobacterium tumefaciens* CFBP6623 (GCA_005221385.1), *Brucella tritici* SY-3 (GCA_023108915.1), and *Ochrobactrum* sp. WY7 (GCA_018437805.1). In contrast, for the *Shinella* acesulfame sulfatase and the ANSA amidase genes, such 100% sequence conservation could not be detected in other bacteria when searching in the NCBI protein nonredundant sequence database. The best match for the *Shinella* acesulfame sulfatase was a 484-aa protein sequence from *Devosia oryzisoli* PTR5 (WP1917753061), which represents a biochemically uncharacterized formylglycine-dependent sulfatase. The amino acid sequence of this *Devosia* sulfatase shares 92% identity at 100% query coverage with the *Shinella* acesulfame sulfatase, while the corresponding nucleotide sequence shows 85% nucleotide identity (at 97% query coverage and 1% gaps in the alignment). Other closely related sequences of uncharacterized formylglycine-dependent sulfatases (>86% protein sequence identity) are present, e.g., in *Devosia nitrariae* NBRC 112416 (WP_284342494.1), *Rhizobium* sp. S152 (WP_289722500.1), and *Paradevosia shaoguanensis* M48 (WP_281736881.1). However, these strains possess only amidase genes that are distantly related to the *Shinella* ANSA amidase gene (e.g., WP_248306280.1 of strain PTR5 with 34.6% amino acid identity at 94% query coverage). Accordingly, their genomes do not possess a sequence with significant similarity (i.e., the local seed 100% alignment is <28 bp as defined by the NCBI megablast default setting) to the *Shinella* ANSA amidase gene. Moreover, the *Shinella* ANSA amidase gene showed high identity (92% at 100% query coverage) only with the ANSA amidase from the previously described acesulfame-degrading *Bosea* and *Chelatococcus* strains,^30^ resulting in 94% identity of the protein sequence.

**Figure 4:**
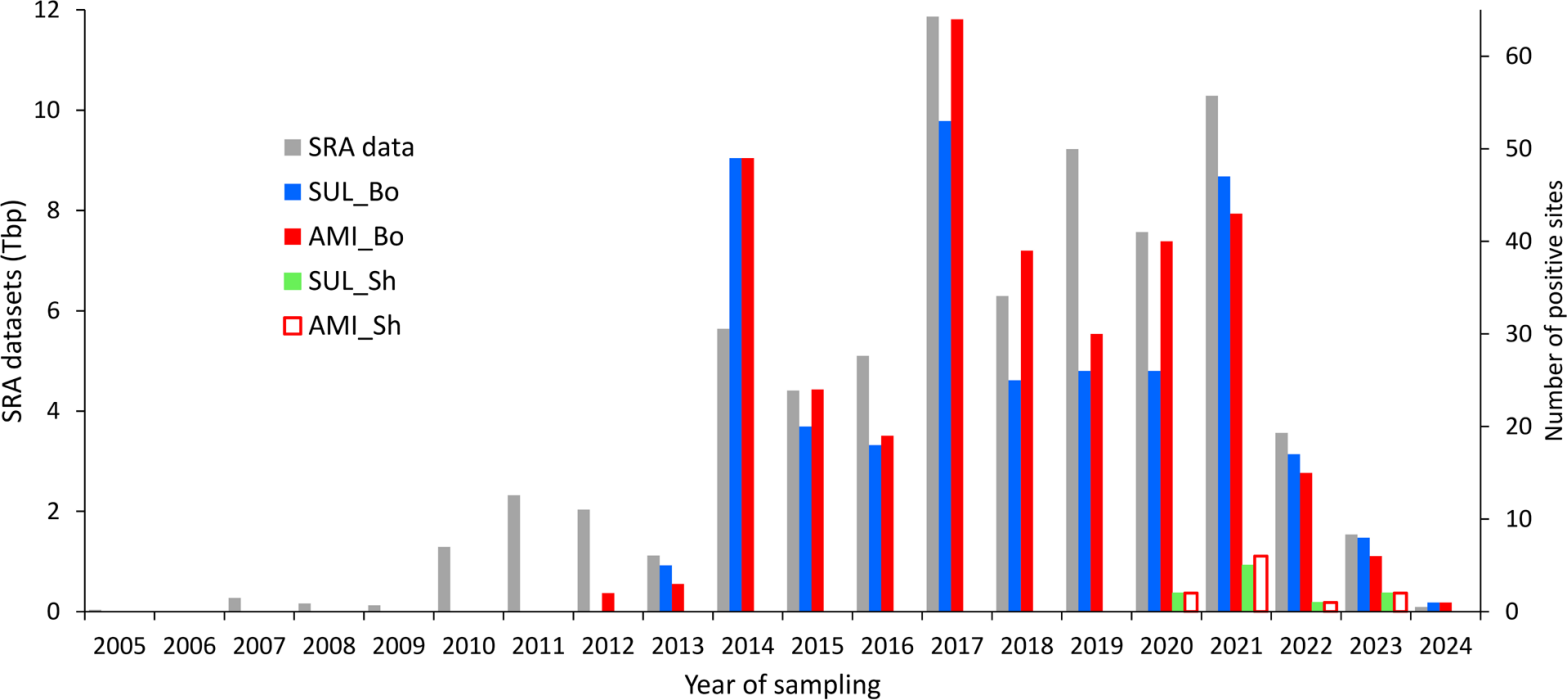
Detection of acesulfame sulfatase (SUL) and ANSA amidase (AMI) gene signatures from the acesulfame-degrading *Bosea/Chelatococcus* (Bo) and *Shinella* (Sh) strains in shotgun metagenome and metatranscriptome SRA data sets related to wastewater, WWTP, or receiving waters across all populated continents. The size of data sets searched by the blastn tool for signatures of the sulfatase and amidase genes as well as the number of positive sites considering sampling campaigns from 2005 to 2024 is shown. For the SUL_Bo and AMI_Bo genes, the number of positive sites directly correlates with the size of SRA data sets searched (see Figure S4).

In summary, even though homologous sequences highly similar to the *Shinella* acesulfame sulfatase and ANSA amidase exist in the databases, identical gene sequences (≥97% identity) were not found when searched with blastp in published genomes and metagenome-assembled genomes (MAGs). Additionally, these *Shinella* genes were not present in any other wastewater-associated metagenome assembly as searched with blastn at NCBI and JGI in this study. Therefore, we extended the blastn search for the *Shinella* acesulfame sulfatase and ANSA amidase genes to SRA files from shotgun metagenome and metatranscriptome sequencing projects related to wastewater, WWTP or receiving waters. In total, more than 6,500 SRA experiment files resulting from sampling campaigns between the years 2005 and 2024 and covering all populated continents were analyzed, amounting to 73.0 tera base pairs (Tbp) of nucleotide sequence data (Table 1, Table S1). In order to compare the occurrence of both the Mn^2+^-dependent and the Mn^2+^-independent acesulfame degradation pathways, we also searched for the *Bosea*/*Chelatococcus* acesulfame sulfatase and ANSA amidase genes. In line with the previous detection of the *Bosea* sp. 100-5 AUP plasmid in several metagenome assemblies,^30^ signatures of the corresponding acesulfame sulfatase and ANSA amidase genes were regularly found in SRA files from most geographical regions (Table 1), albeit often at low sequencing depth that did not allow assembly of complete gene sequences (Figure S3). For all 990 SRA run files with detection of signatures of the four query genes, the median query coverage was only 22% (Table S3). The earliest data sets with *Bosea*/*Chelatococcus* amidase gene signature were from 2012 (Sweden, PRJEB14051, and Hong Kong, PRJNA432264) and with the *Bosea*/*Chelatococcus* sulfatase genes from 2013 (Argentina, PRJNA288131, USA, PRJNA286671 and PRJNA236782, Hong Kong, PRJNA478263, and Luxembourg, PRJNA230567). Both hydrolase genes were almost absent only in samples from Africa and South Asia, which are highly underrepresented in the SRA. Overall, the number of worldwide sampling sites possessing signatures of the *Bosea*/*Chelatococcus* acesulfame sulfatase and ANSA amidase genes correlated with the searched SRA data set size (Figure 4). Signatures of the acesulfame sulfatase and ANSA amidase genes were detected from 2013 on approximately once every 220 giga base pairs (Gbp) of SRA data sets searched. In stark contrast to the abundance of the *Bosea*/*Chelatococcus* sequences, signatures of the *Shinella* acesulfame sulfatase and ANSA amidase genes were rare in the SRA files (Table 1, Figure 4) and could only be found in a couple of data sets resulting from sampling campaigns in China, Mexico, Finland, Spain and Hungary within the years 2020 to 2023 (Table S9). In comparison to the wastewater-related SRA data sets, signatures of both acesulfame pathways were not detected in human gut microbiome SRA data sets (Text S5, Table S2).

**Table 1:** Geographic Distribution and Number of Sampling Sites in Which We Detected Nucleotide Sequences Identical (≥97%) to the Acesulfame Sulfatase (SUL_) and ANSA Amidase (AMI_) Genes From the Acesulfame-Degrading *Bosea*/*Chelatococcus* (Bo) and *Shinella* (Sh) Strains in SRA Data Sets Related to Wastewater, WWTP, or Receiving Waters^a^

				**sites with gene signature detection of**			
**continent**	**data sets (Tbp)**	**campaign period**	**sampling sites**	**SUL_Bo**	**AMI_Bo**	**SUL_Sh**	**AMI_Sh**
Africa	2.6	2013–2022	106	1	0	0	0
West/Central Asia	3.9	2015–2021	48	7	5	0	0
East Asia	24.3	2007–2024	421	122	133	4	5
Southeast Asia	4.4	2010–2021	68	7	10	0	0
South Asia	0.8	2013–2022	48	0	0	0	0
North America	15.7	2005–2023	335	55	66	3	4
South America	1.5	2008–2023	125	8	8	0	0
Europe	18.8	2010–2022	498	92	109	3	2
Oceania	0.9	2008–2019	25	3	4	0	0

a Note: A complete list of all shotgun metagenome and metatranscriptome projects retrieved for the blastn search can be found in Table S1.

## Discussion

Recently, a Mn^2+^-dependent two-step hydrolysis pathway for the degradation of the artificial sweetener acesulfame has been elucidated in *Bosea* and *Chelatococcus* strains isolated from WWTPs.^29,30^ In the current study, we identified and characterized a variation of this pathway in *Shinella* strains isolated from WWTP in which the first step of acesulfame hydrolysis is catalyzed by a novel Mn^2+^-independent, formylglycine-dependent sulfatase instead of the Mn^2+^-dependent MBL-type sulfatase found in *Bosea*/*Chelatococcus*. The subsequent step for the hydrolysis of ANSA to the common metabolite acetoacetate is catalyzed by an amidase signature family enzyme conserved in both pathways.

The enzymes of the acesulfame degradation pathway in *Bosea*/*Chelatococcus* as well as in the *Shinella* strains are encoded by genes located on small plasmids (AUP plasmids) that are annotated to be conjugative and that are about 40 kbp in size. While some diversity among the AUP plasmids has been observed in *Bosea* and *Chelatococcus* strains, e.g., the loss of nonessential genes and rearrangements of larger plasmid segments,^30^ all acesulfame-degrading *Shinella* isolates possess a 100% conserved AUP plasmid. This high conservation might be indicative of a very recent recombination event that gave rise to the *Shinella* AUP plasmid. Although the gene for the formylglycine-dependent sulfatase of the *Shinella* AUP plasmid has not been detected in other bacteria yet, its horizontal transfer to other wastewater populations seems possible, as the gene is independent of complex gene environments and already located in a mobile element. In comparison, the special requirement for Mn^2+^ uptake and selective loading of the MBL-type sulfatase with Mn^2+^ might restrict horizontal gene transfer of the *Bosea*/*Chelatococcus* acesulfame degradation pathway. In this context, it has been reported that species of the genera *Deinococcus*, *Methylobacterium*, *Bosea,* and *Chelatococcus* accumulate high intracellular Mn^2+^ concentrations.^36^

The biochemical function of the *Shinella* formylglycine-dependent acesulfame sulfatase in converting acesulfame to ANSA was confirmed by establishing the enzyme activity in *E. coli* heterologously expressing the *Shinella* gene (SHIWSC3_PJ0001 from *Shinella* sp. WSC3-e). Moreover, for acesulfame-grown *Shinella* sp. WSC3-e, proteomic analysis and activity assays on the SEC-fractionated proteome confirmed the expression of the SHIWSC3_PJ0001 gene and the physiological role of its gene product in hydrolyzing acesulfame. Theoretically, other hydrolases present in the proteome might also contribute to acesulfame degradation *in vivo*. For instance, the SHIWSC3_PF0195 gene was strongly expressed in acesulfame-grown *Shinella* sp. WSC3-e and encodes a CocE/NonD family hydrolase, predicted to catalyze cleavage of esters.^37^ However, acesulfame hydrolysis activity in SEC fractions was only associated with the SHIWSC3_PJ0001 gene product, but not with other hydrolases, indicating that the SHIWSC3_PJ0001 gene product is exclusively responsible for acesulfame hydrolysis *in vivo*. Proteomic analysis also confirmed the high-level expression of the ANSA amidase gene (SHIWSC3_PJ0040) in strain WSC3-e, supporting its role in hydrolyzing ANSA *in vivo*.

Several transport protein candidates encoded in the genome of *Shinella* sp. WSC3-e may be involved in the uptake of the negatively charged acesulfame. One example is the ABC-type transporter encoded by loci SHIWSC3_PJ0026, _PJ0027 and _PJ0028 on the *Shinella* AUP plasmid. However, the encoded proteins were not detected in the proteome of *Shinella* sp. WSC3-e. Alternatively, the highly abundant, chromosome-encoded ABC-type periplasmic binding proteins (encoded by SHIWSC3_4645 and SHIWSC3_3469) together with the corresponding membrane and ATP-binding subunits could be involved in acesulfame import.

After the two-step hydrolysis of acesulfame, the common metabolite acetoacetate is formed, which is likely metabolized via activation to acetoacetyl-CoA and subsequent cleavage to two acetyl-CoA.^28^ The proteome expression pattern of acesulfame-grown *Shinella* sp. WSC3-e is in accordance with a metabolic model in which acetyl-CoA is oxidized via the tricarboxylic acid cycle and in which assimilation proceeds via the glyoxylate cycle, with isocitrate lyase as the key enzyme. In contrast, in strictly anaerobic microorganisms, acetoacetate and acetyl-CoA can be metabolized, e.g., via the Wood-Ljungdahl pathway.^38,39^ Consequently, as the acesulfame-hydrolyzing pathway is also compatible with strictly anaerobic carbon assimilation and dissimilation routes, it seems not to be restricted to the aerobic and facultative denitrifying bacterial strains isolated thus far. However, maturation of the *Shinella* acesulfame sulfatase is dependent on oxygen, as the essential posttranslational modification from the active site cysteine to a formylglycine residue is catalyzed by an oxygenase-like enzyme.^40^ As indicated by the proteomic analysis, the oxygen-dependent SHIWSC3_PF0003-encoded formylglycine-generating enzyme was possibly involved in the sulfatase maturation when strain WSC3-e was grown on acesulfame. For acesulfame mineralization under denitrifying conditions as reported for *Shinella* strains,^31^ the oxygen required for the maturation of the acesulfame sulfatase might be provided during nitrate reduction, as the intermediate nitric oxide can be dismutated to molecular nitrogen and oxygen.^41^ In strictly anoxic environments, alternative pathways (not present in *Shinella*) would be necessary for the sulfatase maturation, e.g., those that employ a radical S-adenosylmethionine formylglycine-generating enzyme for the posttranslational modification of either an active site cysteine or a serine residue to formylglycine.^42,43^

Acesulfame degradation genes were only found at low frequencies in published metagenome and metatranscriptome assemblies related to wastewater environments. This might be due to the inherent limitation of the assembly algorithms: low abundance genes are often discarded by assemblers and, consequently, can be missing in MAGs or other assemblies.^44^ We therefore searched in the SRA database directly and found regular occurrence of the *Bosea*/*Chelatococcus* acesulfame sulfatase and ANSA amidase gene signatures in WWTPs and related environments since 2013.

The earliest shotgun metagenome and metatranscriptome studies dealing with full-scale WWTPs resulted from sampling campaigns around the years 2007 and 2009.^45−47^ Consequently, only a few data sets from this period could be retrieved. However, due to an increasing sequencing effort for wastewater-associated environments afterward, the size of searched SRA sequences related to collection dates in 2010, 2011, and 2012 amounted already to 1.29, 2.32, and 2.04 Tbp, respectively, and covered almost all continents. If the same detection frequency from 2013 on (220 Gbp of SRA data sets searched per detection) would apply for these three years, we would have seen 26 detections of gene signatures for the *Bosea/Chelatococcus* sulfatase and amidase each, but instead only zero and two detections were observed, respectively. This suggests that the corresponding hydrolase genes were either introduced into wastewater habitats later or enriched over time. Interestingly, the detection of *Bosea*/*Chelatococcus* sulfatase and amidase gene signatures in the years 2012/2013 correlates well with the first detection of a substantial acesulfame removal in WWTPs.^23,48^ Such removal appears to be exclusively mediated by the *Bosea*/*Chelatococcus* enzymes, as signatures of the *Shinella* sulfatase and amidase genes could not be detected in samples earlier than 2020. The linear correlation between the number of positive sites for the *Bosea*/*Chelatococcus* genes and the size of the SRA data sets searched indicates that the *Bosea*/*Chelatococcus* pathway has been constantly present since the first detection of the corresponding gene signatures. This finding holds particularly true for wastewater habitats in North America, Europe and East Asia, as other geographical regions are underrepresented in the SRA database. The observed frequency of the acesulfame pathway gene signatures (220 Gbp of SRA data sets searched per detection of *Bosea/Chelatococcus* acesulfame degradation gene signatures) does not represent their real abundance in wastewater. Rather, it is heavily influenced by the average size of the sequencing projects, which depends on the number of samples taken per site and the sequencing depth. For example, SRA experiment files, which typically represent the DNA/RNA sequences of one sample, spanned from about 0.2 Gbp (e.g., PRJDB6962) to almost 600 Gbp (e.g., PRJNA226633).

Due to the low number of recently published SRA data sets, the abundance and distribution of the *Shinella* pathway in wastewater habitats could not be determined unambiguously. SRA files related to metagenome and metatranscriptome projects are typically released with a delay of at least two years due to data processing and preparation of publications. Consequently, only a few data sets resulting from the sampling years 2020/2021 or later are currently available. Nevertheless, the detection since 2020 coincides with the first isolation of acesulfame-degrading *Shinella* strains from WWTPs in Germany in the same year (*Shinella* sp. WSC3-e^30^ and WSD5-1).

Our approach to directly search the raw sequence data of shotgun metagenome and metatranscriptome projects may also be relevant for investigating the dispersal of other genetic markers, e.g., for antibiotics resistance. Accordingly, this search method has recently been applied in a related work for the detection of the trimethoprim resistance gene *drfB* in surface water and wastewater by analyzing 1.7 Tbp of SRA data sets.^49^ As demonstrated now for the *Shinella* hydrolase genes, rare sequences in metagenomes could be missed when only searching in assembled data, e.g., the *Shinella* ANSA amidase gene signature was not found in the MAGs obtained from PRJNA929705 but was detected in the corresponding SRA data set.^50^ Despite their low abundance, these genes might still be of importance in the respective habitat or could become dominant upon enrichment. An example for the enrichment might be the exclusive isolation of acesulfame-degrading *Shinella* strains from reactor experiments seeded with activated sludge from the Sha Tin WWTP in Hong Kong, while the acesulfame degradation genes could not be detected in the MAGs obtained from the same experiment.^31^

The analysis of metagenome data, the monitoring of acesulfame degradation activity in WWTPs^23,48^ and the isolation of acesulfame-degrading bacterial strains are consistent with the appearance of the Mn^2+^-dependent *Bosea*/*Chelatococcus* acesulfame degradation pathway not before the year 2012. Already after 2012, however, the respective sulfatase and amidase genes could be regularly detected in samples from wastewater treatment systems around the world, indicating a fast and unhindered spreading of the pathway. In line with this, the Mn^2+^-dependence of the pathway appears not to be a limitation, as manganese is a relatively abundant heavy metal in municipal wastewater due to various anthropogenic sources, such as ubiquitous steel abrasion and corrosion.^51^ Therefore, the detection of gene signatures of an alternative Mn^2+^-independent acesulfame sulfatase about seven years later than the *Bosea*/*Chelatococcus* enzyme is somewhat surprising. Interestingly, both pathways may coexist, as shown for sites in Mexico, Finland and China. However, a temporary Mn^2+^ depletion in WWTPs, e.g., due to a microalgal bloom^52^ or when treating wastewater with microalgae as in projects PRJNA807808,^53^ PRJNA983699 and PRJNA970524, might have facilitated the enrichment and maintenance of the Mn^2+^-independent pathway.

In conclusion, at least two different hydrolase mechanisms for acesulfame exist despite the catalyzed nucleophilic attack toward the sulfur atom is challenging due to acesulfame’s electron-rich ring system.^54^ Both the Mn^2+^-dependent and the Mn^2+^-independent sulfatases showed nature’s potential to provide efficient catalysts for highly challenging chemical reactions. To evaluate the ecological impact of the identified acesulfame sulfatases and ANSA amidases, a detailed biochemical characterization might be promising to elucidate their substrate specificity and the underlying structural determinants. Whether exclusively the two identified pathways or also others are responsible for acesulfame degradation in real wastewater can now be investigated by quantitative PCR for comparing copy numbers of the known acesulfame degradation genes with the degradation efficiency in WWTPs. This approach could be complemented by metagenome, metatranscriptome and metaproteome analyses. Furthermore, our approach of searching for gene signatures directly in the SRA data could be extended to other environments for discovering the evolutionary relationships between the acesulfame degradation genes as well as their global distribution.

## Supporting Information Available

Genome sequencing of strain WSD5-1 (Text S1); shotgun proteomics (Text S2); blastp search in the NCBI protein database and blastn search in assembled nucleic acid sequence databases (Text S3); detailed description of SRA data set compilation and criteria for blastn search (Text S4); blastn search in additional data sets (Text S5); comparison of *Bosea/Chelatococcus* and *Shinella* ANSA amidase gene sequences (Text S6); comparison of the *Shinella* acesulfame sulfatase gene sequence with its closest match in the NCBI database (Text S7); sulfatase-maturating enzymes in the *Shinella* genomes (Text S8); alignment of the *Shinella* sp. YE25 genome against the *Shinella* sp. WSC3-e genome (Figure S1); dendrogram of hierarchical cluster analysis for acesulfame degradation and abundance distribution of 200 most abundant *Shinella* sp. WSC3-e proteins after summing up the proteins in 30 SEC fractions (Figure S2); examples for blastn results with acesulfame sulfatase genes from acesulfame-degrading *Bosea*/*Chelatococcus* and *Shinella* strains as query against data sets from the SRA database (Figure S3); historic occurrence of *Bosea*/*Chelatococcus* acesulfame sulfatase (SUL_Bo) and ANSA amidase (AMI_Bo) gene signatures in shotgun metagenome and metatranscriptome SRA data sets related to wastewater, WWTP, or receiving waters across all populated continents (Figure S4) (PDF)
Searched shotgun metagenome/metatranscriptome projects related to wastewater, wastewater treatment, and wastewater receiving waters (Table S1); searched shotgun metagenome/metatranscriptome projects related to human gut microbiome (Table S2); query gene coverage of read alignments per SRA run file (Table S3); genome comparison of acesulfame-degrading strains *Shinella* sp. WSC3-e, WSD5-1, and YE25 (Table S4); coding sequences of the AUP plasmid from *Shinella* sp. WSC3-e with predicted functions (Table S5); 20 most abundant proteins in *Shinella* sp. WSC3-e when growing with acesulfame (Table S6); identified proteins in the crude extract proteome of *Shinella* sp. WSC3-e (Table S7); 200 most abundant proteins found in SEC fractions (Table S8); SRA data sets of metagenome projects with detection of the *Shinella* acesulfame sulfatase and ANSA amidase gene (Table S9) (XLSX)
