# Supplementary material for: A Novel Sulfatase for Acesulfame Degradation in Wastewater Treatment Plants as Evidenced from Shinella Strains
Source: Environ Sci Technol. 2024 Oct 7;58(42):18892–902. doi: 10.1021/acs.est.4c02283 (PMC11500405; doi:10.1021/acs.est.4c02283)
Supplement: Supplementary file 1 — es4c02283_si_001.pdf [file es4c02283_si_001.pdf]

Supporting Information

A novel sulfatase for acesulfame degradation in wastewater treatment plants as evidenced from *Shinella* strains

Yu Liu[1,2], Thore Rohwerder[3,*], Maria L. Bonatelli[3], Theda von Postel[1,2], Sabine Kleinsteuber[3], Lorenz Adrian[1,2], Chang Ding[1,*]

[1]Helmholtz Centre for Environmental Research – UFZ, Molecular Environmental Biotechnology, Leipzig, Germany
[2]Chair of Geobiotechnology, Technische Universität Berlin, Berlin, Germany
[3]Helmholtz Centre for Environmental Research – UFZ, Microbial Biotechnology, Leipzig, Germany

Total pages: **13;** total figures: **4;** total supplementary text: **8**

Text S1. Genome sequencing of strain WSD5-1 2
Text S2. Shotgun proteomics 2
Text S3. Blastp search in the NCBI protein database and blastn search in assembled nucleic acid sequence databases 3
Text S4. Detailed description of SRA dataset compilation and criteria for blastn search 4
SRA database 4
Compilation of wastewater-related SRA datasets for blastn search 4
SRA blastn search criteria 5
Text S5. Blastn search in additional datasets 5
Blastn search in SRA datasets related to human gut microbiome 5
Text S6. Comparison of *Bosea/Chelatococcus* and *Shinella* ANSA amidase gene sequences 6
Text S7. Comparison of the *Shinella* acesulfame sulfatase gene sequence with its closest match in the NCBI database 7
Text S8. Sulfatase-maturating enzymes in the *Shinella* genomes 8
Genes encoding aerobic sulfatase maturation 8
Genes encoding anaerobic sulfatase maturation 8
Supplementary Figures 9
References 13

Figure S1. Alignment of the *Shinella* sp. YE25 genome (green and turquoise) against the *Shinella* sp. WSC3-e genome (gray). 9
Figure S2. Dendrogram of hierarchical cluster analysis for acesulfame degradation and abundance distribution of 200 most abundant *Shinella* sp. WSC3-e proteins after summing up the proteins in 30 SEC fractions. 10
Figure S3. Examples for blastn results with acesulfame sulfatase genes from acesulfame-degrading *Bosea*/*Chelatococcus* and *Shinella* strains as query against datasets from the SRA database. 11
Figure S4. Historic occurrence of *Bosea*/*Chelatococcus* acesulfame sulfatase (SUL_Bo) and ANSA amidase (AMI_Bo) gene signatures in shotgun metagenome and metatranscriptome SRA datasets related to wastewater, WWTP or receiving waters across all populated continents. 12

Text S1. Genome sequencing of strain WSD5-1

For DNA extraction, *Shinella* sp. WSD5-1 was grown on 5 mM acesulfame in mineral salt medium (DSMZ 461) at 30°C until late logarithmic phase. DNA was extracted with MagAttract HMW DNA Kit (QIAGEN, Hilden, Germany), according to the manufacturer's instruction. DNA integrity was checked by gel electrophoresis, while DNA concentration was measured with the Qubit dsDNA BR Assay Kit (Thermo Fisher Scientific, Rockford, IL, USA). The genome was sequenced with both long- and short-read techniques. Long-read sequencing was performed on the MinION Mk1B platform (Oxford Nanopore Technologies, Oxford, United Kingdom) with a library prepared using the ligation sequencing kit V14 (SQK-LSK114). Sequencing was done using a flow cell R10.4.1 with the software MinKNOW UI v.23.07.15. Basecalling was performed with dorado v.0.4.2 (Oxford Nanopore Technologies), and filtlong v.0.2.1 (github.com/rrwick/Filtlong) was used to filter out the 40% worst reads. Short-read sequencing was done on the Illumina NovaSeq platform (2 × 150 bp; Azenta, Leipzig, Germany). Assembly was conducted with flye v.2.9.2,[1] and genome polishing was done with pilon v.1.23.[2] All software tools were used with default parameters. Genome annotation, classification and quality measurements were done as previously described.[3]

Text S2. Shotgun proteomics

Shinella sp. WSC3-e crude extract and SEC fractions were analyzed using shotgun proteomics. Each SEC fraction subsample (70 µL) was spiked with 4 µL glyceraldehyde-3-phosphate dehydrogenase (GAPDH, from *Staphylococcus aureus* MRSA252, NCBI ID: WP_000279414, 336 aa residues) as the internal standard to account for sample to sample variation among fractions. Purified GAPDH (concentration between 16 and 20 mg mL^{-1}) was diluted 1,000 times in water before usage. Crude cell extract (120 µL, 0.33 µg protein μL^{-1}) was processed directly in the following steps without adding GAPDH.

An equal volume of sodium deoxycholate 10% (w/v) was added to all samples in order to denature proteins. Proteins were reduced with 12 mM DTT in 45 min at 37°C and then alkylated with 40 mM iodoacetamide in 45 min at room temperature.[4] Before trypsin digestion, samples were diluted with 100 mM ammonium bicarbonate to a final concentration of 1% (w/v) sodium deoxycholate. Trypsin digestion was done by adding 5 µL reductively methylated trypsin (Promega, Madison, USA) to each sample and incubating the samples overnight at 37°C. Digestion was stopped by adding neat formic acid to a final concentration of 2% (v/v). Precipitated material was carefully removed by centrifugation twice at 16,000 × *g* and 4°C for 10 min. Digested samples were desalted by using 100-µL C_{18} ziptips (Pierce™, Thermo Fisher Scientific, Massachusetts, USA) and measured on a nano-LC-MS/MS (Orbitrap) as described previously.[5]

Protein identification was conducted using the Proteome Discoverer 2.4 (Thermo Fisher Scientific, Massachusetts, USA) applying the SequestHT search engine with the protein database of *Shinella* sp. WSC3-e. Other parameter settings were as described previously[5] except that mass tolerance for fragment ion was reduced from 0.5 Da to 0.1 Da. Protein and peptide abundance values were calculated by intensity-based label free quantification using the Minora node implemented in Proteome Discoverer.[5] Protein quantities were normalized using the GAPDH intensity in each SEC sample. Hierarchical cluster analysis was done in OriginPro, Version 2023 (OriginLab Corporation, Northampton, Massachusetts,

USA). Correlation was applied to cluster variables into five clusters. Group average was set as the linkage method to calculate the distance among clusters. Clustroid was found by using the sum of distances measured from all other variables in the cluster. The hierarchical tree was shown in a dendrogram plot.

Text S3. Blastp search in the NCBI protein database and blastn search in assembled nucleic acid sequence databases

Blastp search at NCBI

The NCBI blastp tool was used to search for amino acid sequences related to the acesulfame sulfatase (SHIWSC3_PJ0001 gene product) and ANSA amidase (SHIWSC3_PJ0040 gene product) from *Shinella* sp. WSC3-e against the NCBI non-redundant protein sequence database (July 2024). This approach covers all annotated genomes and metagenome assemblies deposited at NCBI (i.e., all translated GenBank coding sequences) plus protein sequences deposited at PDB, SwissProt, PIR and PRF and excludes other sequences of whole genome shotgun projects from environmental samples (see blastn search below).

Blastn search at NCBI and JGI

Other assembled nucleic acid datasets not yet analyzed for open reading frames (e.g., partial assemblies or permanent drafts) were searched with blastn (megablast, NCBI default settings, without filters and masks). For doing this, datasets from whole genome shotgun sequencing, metagenome and metatranscriptome projects in the NCBI whole genome shotgun contigs database and the JGI database (July 2024) originating from wastewater, activated sludge or bioreactor samples were searched for sequences similar to the *Shinella* acesulfame sulfatase (SHIWSC3_PJ0001) and ANSA amidase (SHIWSC3_PJ0040) genes. At NCBI, the blastn search was limited to the following metagenomes: activated sludge (taxid:942017), aquatic (taxid:1169740), aquifer (taxid:1704045), bioreactor (taxid:1076179), bioreactor sludge (taxid:412754), drinking water (taxid:2651591), freshwater (taxid:449393), groundwater (taxid:717931), lagoon (taxid:1763544), lake water (taxid:1647806), pond (taxid:1851193), sludge (taxid:1592332) and wastewater (taxid:527639). At JGI, blastn search was done in the metagenome and metatranscriptome datasets wastewater treatment plant, wastewater and bioreactor.

The NCBI whole genome shotgun contigs database (organism: Alphaproteobacteria) was used to search for the whole 41,586-bp plasmid (OZ000540) bearing the *Shinella* acesulfame sulfatase and ANSA amidase genes (blastn, megablast with default settings).

Pairwise comparison with blastp and blastn

For pairwise comparison of related proteins and genes, sequences were aligned with the NCBI blastp and blastn tools, respectively, with default settings to obtain query coverage and identity values.

Text S4. Detailed description of SRA dataset compilation and criteria for blastn search

SRA database

The Sequence Read Archive (SRA) at NCBI is a publicly available repository of raw sequencing data. In this database, amplicon and shotgun nucleic acid sequence reads from metagenome and metatranscriptome projects as well as from genome sequencing projects of microbial strains and other organisms from all branches of life can be found. Most metagenome and metatranscriptome sequencing projects employed various Illumina platforms, which generate short reads between 50 and 500 bp, and the predominant reads in the database are Illumina 150-bp reads. Besides, a few datasets were generated by other short-read sequencing methods (Ion Torrent, Sanger and Roche 454) or by long-read sequencing technologies (PacBio and Oxford Nanopore).

Compilation of wastewater-related SRA datasets for blastn search

The NCBI BioProject, BioSample and SRA databases were searched for wastewater-related shotgun sequencing metagenome and metatranscriptome projects using the keywords “wastewater”, “sewage” and “sludge”. In addition, BioProject accession numbers provided in publications dealing with wastewater metagenome and metatranscriptome studies were also considered. Datasets compiled for the blastn search cover raw sewage, full-scale WWTPs (influent, effluent and all treatment steps, such as aerobic activated sludge, anammox, comammox and anaerobic digester), constructed wetlands treating wastewater, environments receiving treated or untreated wastewater (e.g., rivers, lakes, lagoons), pilot-scale plants and lab reactor experiments seeded with material from WWTPs and/or treating real wastewater.

We excluded datasets when the year of sample collection or the number of sampling sites are not provided. Datasets were also excluded when only related to wastewater of industrial origin and corresponding WWTPs that only treat, e.g., acid mine drainage, oil sand wastewater, brewery wastewater, agricultural waste, textile industry waste, coking wastewater or paper mill wastewater. Datasets related to lab experiments with synthetic wastewater, enrichment cultures or microbial isolates were excluded as well. Furthermore, datasets with DNA/RNA extraction methods not allowing or substantially reducing the recovery of bacterial nucleic acid (e.g., only extracting DNA/RNA from supernatant after centrifugation or from filtrate after 0.2-µm filtration) were not considered.

SRA blastn search

Raw sequence files of shotgun metagenome and metatranscriptome projects in the context of wastewater environments were downloaded from the NCBI SRA (Table S1) and searched with blastn (megablast, NCBI default settings, without filters and masks) at the high-performance-computing cluster EVE (UFZ/iDiv) using the acesulfame sulfatase and ANSA amidase genes from *Bosea*/*Chelatococcus*[3] and *Shinella* as query. In addition to the wastewater-associated SRA datasets, SRA files related to human gut microbiome (Table S2) were downloaded and searched with blastn for acesulfame sulfatase and ANSA amidase gene signatures (see Text S5).

Query gene coverage of read alignments per SRA run file was calculated by subtracting overlapping sequences of the aligned reads (Table S3).

SRA blastn search criteria

The criteria for detecting signatures of the *Bosea*/*Chelatococcus* and *Shinella* acesulfame sulfatase and ANSA amidase genes in SRA datasets with blastn (alignment of ≥140 bp showing ≥97% identity) were mainly oriented by the error rates of the prevailing sequencing technologies employed and the predominant read length (150 bp) present in the SRA database. The error rates depend on the sequencing technology and span for short-read sequences from 0.1-1% for Illumina[6-8] to 1.1% for Roche 454[9] and about 2% for Ion Torrent.[10] In contrast, long-read sequencing platforms typically display higher error rates ranging from 10 to 20%,[11] particularly, due to the occurrence of insertions and deletions. Therefore, in order to consider most of the short-read datasets, we set the threshold for detection to 97% identity. As the predominant read length is not always exactly 150 bp but shows some variation, we set the required alignment length with the query gene to ≥140 bp.

These search criteria are highly stringent, as they are able to distinguish highly similar homologous sequences such as the *Shinella* acesulfame sulfatase gene vs the *Devosia oryzisoli* PTR5 sulfatase gene WP_191775306.1 (84.8% nucleotide sequence identity) and the two ANSA amidase genes from *Bosea*/*Chelatococcus* and *Shinella* (outlined in Text S6 and Text S7). Likewise, the criteria for partial alignments (matching ≥30 bp at 97% identity at start or end regions of query genes and matching ≥140 bp at 97% identity including flanking sequences) are highly specific.

Text S5. Blastn search in additional datasets

Blastn search in SRA datasets related to human gut microbiome

Besides the SRA datasets related to wastewater environments (as defined above), we searched also for the presence of *Bosea*/*Chelatococcus* and *Shinella* acesulfame sulfatase and ANSA amidase gene signatures in a compilation of SRA files related to human gut microbiome. These gut microbiome datasets comprise samples from East Asia, North America and Europe with collection years 2011 to 2023 (Table S2). In total, 52.6 Tbp were searched without any detection of the four query genes (alignment of ≥140 bp showing ≥97% identity).

Text S6. Comparison of *Bosea/Chelatococcus* and *Shinella* ANSA amidase gene sequences

The closest relative of the *Shinella* ANSA amidase in the NCBI non-redundant protein database is the *Bosea/Chelatococcus* ANSA amidase. When aligned with blastn, the corresponding gene sequences (both 1419 bp) share 92.3% identical nucleotides (1310/1419 bp) resulting in a total number of 109 mismatches for the complete alignment. A closer inspection of the sequence alignment with CLUSTAL Omega (https://www.ebi.ac.uk/jdispatcher/msa/clustalo) revealed that both genes are clearly distinguishable at a sub-alignment length of ≥140 bp, as the maximal indistinguishable sub-alignment length (i.e., showing 100% identity) is only two times 53 bp and two times 55 bp (highlighted in yellow and green). Sub-alignments that meet our blastn search criteria of ≥136/140 bp identity (≥97%) can only be found in the 164-bp region spanning from position 94 to 257 (highlighted in blue and gray). Within this region, only eight alignments show exactly 136/140 bp identity (97.1%). Consequently, in these eight cases, an alignment with both the *Shinella* and *Bosea/Chelatococcus* ANSA amidase gene as query meets our SRA search criteria (≥140 bp with ≥97% identity). However, as no sub-alignment shows >136/140 bp identity (>97.1%), the direct comparison of the blastn results for the two amidase genes always allows to distinguish between *Shinella* and *Bosea/Chelatococcus* gene signatures in the SRA datasets searched.

```
AMI_Bo      ATGACATCCATCGACGAGATTACGAGGAAGCCTGCACAGGTATTAGCCGGGCTGATTCGCGAGCGCGAGCTATCATGCCTGGAAGTTACCTCGGCTTTCTTGAAAAGGATCGATGACATC  120
AMI_Sh      ATGACGTGTATCGACGAGATTACGACGAAGCCTGCACAGGTATTAGCCGGGTTGATTCGCGAGCGCGAGCTCTCATGCCTGGAAGTTACATCCGCTTTCTTGAAAAGGATCGATGATATC  120
            *****.*..****************.*************************.*******************.*****************.**.***********************.***
                                                                                                             ***********************.***

AMI_Bo      AATCCAAAGATAAATGCATTTTGTACCGTCCTACACGAAGCGGCGCTCGCTGCGGCGGCGCAAGCCGATCAAGCCTTCACAAGCGGTTCTCCGATCGGACCGCTCCACGGCCTGCCGGTG  240
AMI_Sh      AATCCAAAGATAAATGCATTTTGCACCGTCCTACACGAAGCGGCGCTCGCTGCGGCGGCGCAAGCCGATCAAGCCTTCAGAAGCGGGTCTCCGATCGGACCGCTCCACGGCCTGCCGGTA  240
            ***********************.*******************************************************.******.********************************.
            ***********************.*******************************************************.******.********************************.
            ***********************.*******************************************************.******.********************************.

AMI_Bo      GCGCTCAAGGATTTGACCCCGACTAAGGGAGTTAGGACCACGCGCGGCTCGCGTCTTTTCGAAAACGCCGTGCCAGCTGAGGACGCTGAGCTCGTCAGGAGGCTCAAGAAAGCCGGAGCC  360
AMI_Sh      GCGCTCAAGGATTTGACACCGACTAAGGGAGTTCGAACCACCCGCGGCTCACGTCTTTTCGAAAACGCCGTGCCAACTGAGGACGTTGAACTCGTCAGGAGGCTCAAGAAAGCCGGAGCC  360
            *****************.***************.*.*****.********.************************.*********.***.******************************

AMI_Bo      ATTGTCATCGGCAAAACAAATACACCGGAGTTTGGGCATAAAGGCGAGACGGATAATCTCATTTTCGGCCCGACGCGCAACCCGTGGCGTCTCGATCGCACTCCTGGTGGATCGAGTGGG  480
AMI_Sh      ATTCTCATTGGCAAAACAAATACTCCGGAGTTTGGGCATAAAGGCGAGACCGATAATCTCATTTTCGGCCCGACTCGCAACCCGTGGCGTCTTGATCGGACTCCTGGCGGATCGAGTGGC  480
            ***.****.**************.**************************.***********************.*****************.*****.********.***********.

AMI_Bo      GGCTCAGCCGCTGCCGTAGCTGCGGGATTGGTTCCGTTTGCAGAGGGTAGCGATGGTGCGGGATCGATCCGAATCCCGGCGAGCATGTGCGGAATCTTCGGCTTCAAACCAAGCTACGGG  600
AMI_Sh      GGCTCAGCAGCTGCTGTAGCTGCGGGATTGGTCCCGTTTGCAGAGGGTAGCGATGGCGCGGGATCGATCCGAATCCCGGCGAGTATGTGCGGAATCTTCGGCTTCAAACCAAGCTACGGG  600
            ********.*****.*****************.***********************.**************************.************************************

AMI_Bo      CGAGTGCCCGATGTCGCTGGCCCATTCTCCAGCCACACTCCGTTCTTCCACAATGGTCCCCTGGCGCGAAGCGTGGGCGATGCCACGCTCCTATATCAGGCGATGGTTGGAGCCGACAGC  720
AMI_Sh      CGAGTGCCCGATGTCGCCGGTCCGTTCTCCAGCCACACTCCATTTTTCCACAATGGTCCCCTGGCGCGAAGCGTGGGCGATGCCGCGTTACTGTATCAGGCGATGGTCGGAGCCGACAGC  720
            *****************.**.**.*****************.**.***************************************.**.*.**.**************.************

AMI_Bo      GCTGATCCGTTCTCCGTTCCGACTGACCAGGATGTGCTGATGTCTCTTGACCACGGGGTTGCCGGCCTGCGCGTGGCATTCAGCGTGAATTTGGGGTACTTTGAGGTGTCCGATGAGGTG  840
AMI_Sh      TCCGATCCGTTTTCCGTCCCAGCCGACCAGGATGCGCTGATATCTCTTGACCACGGTGTTGCCGGCCTGCGAGTGGCATTCAGCATGAATTTGGGATATTTTGAGGTGTCCGATGAGGTG  840
            .*.********.*****.**..*.**********.******.**************.**************.************.**********.**.*********************

AMI_Bo      AAACTAGCCTGTACAAGGGCCACGGAGGCGTTTGCGGCGCTAGGTTGCGTCGTCGACGAGGTGGAGGTGGACTTCGACCGTGAATTGGAGGCGGCTTTCTTCACCCTCTGGTGTGCAAAG  960
AMI_Sh      AAGCTAGCCTGTACGCGGGCCACAGAGGCGTTTTCGGCGCTAGGTTGCGTCGTTGACGAGGTGGAGGTGGGCTTCGACGGTGACTTAGAGGCGGCTTTCTTCACCCTGTGGTGCGCAAAG  960
            **.***********..*******.*********.*******************.****************.*******.****.**.********************.*****.******

AMI_Bo      TTGGCGACTGTCTATTCCAACATCACGGACAGCGAATTCTCGTTGCTTGAGCCAGTGGTTCAGGGCCTGATCGAACAAGGCCGGCGATTGAGTGCCGTCGAATTTGGCCGCGCAAATCTC  1080
AMI_Sh      TTGGCGACGGTCTATTCCGGCATTACGGACCGCGAATTCTCGATGCTGGAGCCGGTGGTTCAGGGCCTGATCGAACGAGGCAGAGAATTGAGCGCCGTCGAGTTTGGCCGGGCAAATCTC  1080
            ********.*********..***.******.***********.****.*****.**********************.****.*...******.********.********.*********

AMI_Bo      ATGCGCGAAGTCGTTTGGAGTAGGCTGTGTTCTATTTTTGACAAGTATGATGTGTTGATCTGCCCAACAACAGCAGTTTCCGCCTTCCCTATTGAGAATGGGCCACCAGCTACGATCAAC  1200
AMI_Sh      GTCCGCGAAGTCGTTTGGGGCAGGCTGTGCTCTATTTTTGAGAAGTATGATGTGTTGATCTGCCCAACAACAGCGGTTCCCGCCTTTTCTATAGAGAATGGGCCACCCGGTACGATCAAC  1200
            .*.***************.*.********.***********.********************************.***.*******..****.**************.*.**********

AMI_Bo      GGCGCGTCGATCAATCGCCTTCTGGGCTGGTTCCTGACTTATCCCTTCAACTTCACAGGAAATCCTGCCGCCTCCGTTCCTTGTGGATTTTCTCATGACGGCCTCCCAATCGGGATGCAG  1320
AMI_Sh      GGCGCGTCGATTAATCGCCTTCTGGGCTGGTTCCTGACTTATCCCTTCAACTTCACAGGAAATCCCGCCGCATCCGTTCCGTGTGGATTTTCTCATGACGGCCTCCCGATCGGGATGCAG  1320
            ***********.*****************************************************.*****.********.**************************.************

AMI_Bo      ATTATCGGAAGGCGCCTCGATGACGGCTTGGTTTTACGAGCTTCCCGCACGTTCGAGCGTCTATCTCCTTGGCCCAAACTAGCAAATCCAAGCTTTTAA    1419
AMI_Sh      ATTATCGGAAGGCGTCTCGATGACGGCTTGGTTTTGCGCGCTTCCCGCACGTTCGAGCGTCTATCTCCTTGGCCCAAACTAGCAAATCCAAGCTCTTAA    1419
            **************.********************.**.*******************************************************.****
```

CLUSTAL Omega (1.2.4) sequence alignment of complete *Bosea/Chelatococcus* and *Shinella* ANSA amidase genes (AMI_Bo and AMI_Sh, respectively). Symbols: * identical; . mismatch (highlighted in red); all sub-alignments with 53/53 bp identity (100%), with 55/55 bp identity (100%), with 142/146 bp identity (97.3%) including 7 x 136/140 bp (97.1%) and with 159/164 bp identity (97.0%) including 1 x 136/140 bp (97.1%) are shown.

Text S7. Comparison of the *Shinella* acesulfame sulfatase gene sequence with its closest match in the NCBI database

The closest relative of the *Shinella* acesulfame sulfatase in the NCBI non-redundant protein database is the *Devosia oryzisoli* PTR5 sulfatase WP_191775306.1 (uncharacterized predicted protein). When aligned with blastn, the corresponding gene sequences (1509 bp for the *Shinella* acesulfame sulfatase gene, 1455 bp for the *Devosia oryzisoli* PTR5 sulfatase WP_191775306.1 gene) share 84.8% identical nucleotides (1251/1476 bp). A closer inspection of the sequence alignment with CLUSTAL Omega revealed that both genes are clearly distinguishable at a sub-alignment length of ≥140 bp, as the maximal indistinguishable sub-alignment length (i.e., showing 100% identity) is only once 38 bp and once 40 bp (highlighted in yellow and green). The best sub-alignments of ≥140 bp show only 90.3% identity (highlighted in blue and gray).

```
SUL_Sh          ATGACGATTTCGAGAAATTGCGCGACTGGGGTTATCATGACCAAACCCAACATCTTGTGGATATGCACTGACCAGCAGCGCTGGGACACGCTTGGCATTACCGGCAATCGCCATGTGCGA 120
SUL_Dev         ------------------------------------ATGGCCCAGCCAAACATCTTGTGGATCTGCACCGACCAGCAACGCTGGGATACCTTGGGCGTCACCGGCAACCGCCATGTGCGA 84
                ....................................***.**.*.**.**************.*****.********.********.**..*.***.*.********.************

SUL_Sh          ACTCCGGTGCTCGACAAGCTCTTCGGCGAGGCAGCCTGGTTCTCCGGGGCCTATTGCCAGTCGCCGGTCTGCACGCCCAGTCGCGCGAGCTTTCTCACCGGCCGCTATCCGCGTACGACC 240
SUL_Dev         ACCCCGGTGCTCGACAAGCTCTTTGCCGATGCGGCGTGGTTCTCCGGCGCTTACTGCCAGTCCCCGGTCTGCACCCCGAGCCGCGCCAGTTTCCTCACGGGGCGCTATCCGCGCACCACC 204
                **.********************.*.***.**.**.***********.**.**.********.***********.**.**.*****.**.**.*****.**.***********.**.***

SUL_Sh          CGGACGCGCCAGAACGGGCAGGACATGCCGGCCGACGAAATGCTGGTGACGCGGCTGCTCGCCGAGAATGGCTATACCTGCGGTCTATCGGGCAAGCTCCATATCTCGGCGGCCAATCCG 360
SUL_Dev         CGAACCCGCCAGAACGGGCAGGACATGCCGCCCGACGAAATGCTGGTCACGCGGCTCCTGGCCGACAACGGCTATACCTGCGGCCTGTCGGGCAAGCTGCACATCTCGGCGGCCAATCCC 324
                **.**.************************.****************.********.**.*****.**.**************.**.***********.**.*****************.

SUL_Sh          AGCGTGGCGCCGGTCATCGAACCGCGCATCAATGATGGCTATGCCGCCTTTCACTGGTCGCATCATCCGGGTTGTCGGATGGTACTTAATGACTACGGCAGCGCGCAGAACTGGCCACTC 480
SUL_Dev         AGCGTGGCGCCGGTCAGCGAGCCGCGCATCAACGATGGGTACACGGCGTTTCACTGGTCGCATCACCCGGG------------------ACACTACGGCAGCGCGCAAAACTGGCCGCTC 426
                ****************.***.***********.*****.**..*.**.*****************.*****....................****************.********.***

SUL_Sh          AACGAATACAATCTATGGCTGCTCGAGCGCGGCGCCGAATACCGGGCTGACCCCTATCGGGGCTCCAGATATGTGAAGGCGGGGCCCGAGACAAAGAACCACCAGACCACCTGGTGCGCC 600
SUL_Dev         AACGAATACAACCTCTGGCTGCTCGAGCGCGGCGCTGATTACACCGCCGACCCCTATCGCGGCTCGAAATATGTCAAGGCCGGCCCCGAGGCGCAGAACCACCAGACCACCTGGTGCGCC 546
                ***********.**.********************.**.***...**.***********.*****.*.******.*****.**.******.*..**************************
                                                                                                          **************************

SUL_Sh          GACAAGGCCATCAATTTCGTCAAGGCGCATGAGGCGAACGACCGGCCCTGGCTGTTCTCGGTCAACTTCTTCGACCCTCATCACCCCTTCGATCCGCCCGCCGAGTATCTCGATCGCTAC 720
SUL_Dev         GACAAGGCCATCAACTTCGTCAAGGCGCATCAGGGCGCCGGCCGGCCCTGGCTGTTCTCGGTCAATTTCTTTGACCCGCATCACCCCTTCGATCCGCCAGCCGAATATCTTGACCGCTAT 666
                **************.***************.***....**.************************.*****.*****.********************.*****.*****.**.*****.
                **************.***************.***....**.************************.*****.*****.********************.*****.*****.**.*****

SUL_Sh          CTCGACCGGCTCGACGAGATCGAGTTGCCGAACTACCGGCCGGGAGAGCTGGAAGAAAAACCTGCCTTCCAGAAGATCGACCACCAGGGAGCCTATGGCGGCAATGCCGGCATGCCCTAC 840
SUL_Dev         CTGGACCGGCTCGATGAGATCGAACTGCCCAATTACCGGCCGGGTGAACTCGAGGACAAGCCCGCCTTCCAGAAGATCGACCATGAGGGTGCCTATGGCGGCAATGCCGGGATGTCCTAT 786
                **.***********.********..****.**.***********.**.**.**.**.**.**.********************..****.********************.***.****.

SUL_Sh          GACGAGATGACCGATCATGACCACAGGCTGGTGCGCGCGGCTTATTTCGCCATGTGCGACTTGATCGATGACCAGGTCGGCCGCATGCTCGACGCCCTGCGCGATACCGGTCAGCTCGAC 960
SUL_Dev         GACGAGATGACTGACCACGACCACCGGCTGGTACGGGCCGCCTATTTCGCCATGTGCGATCTCATCGACGACCAGGTGGGCCGCATGTTCGAGGCGCTGCGCGAGACCGGCCAGCTCGAC 906
                ***********.**.**.******.*******.**.**.**.*****************..*.*****.********.*********.****.**.********.*****.*********

SUL_Sh          AATACCATCGTCATCTTCATGTCCGACCACGGCGAGCTGCTGGGCGATCATGGCGTCTATCTCAAGGGGCCGTTCTTCTACGAGCCCAGCGTCCATGTTCCCCTGGCGATCTCGTGGCCG 1080
SUL_Dev         AACACCATCGTCATCTTCATGTCCGACCACGGCGAACTGCTCGGTGACCACGGGGTCTACCTCAAGGGGCCGTTCTTCTACGAACCCAGCGTCCATGTTCCACTCGCGATCTCCTGGCCG 1026
                **.********************************.*****.**.**.**.**.*****.***********************.*****************.**.********.******
                                                                      *****.***********************.*****************.**.********.******

SUL_Sh          GGCCATATTCCGGCCCGCCGAATCGACGGGCTGGTCGAACTGACCGATATCGCGCCGACCCTGCTCGAGGCGGCGGGGCTCGATATCTGGCCGGGCATGCAGGGCCGCTCGCTCTGGCCG 1200
SUL_Dev         GGGCACATTCCCGCCCGCCGCATCGATGGGCTGGTGGAACTGGTCGACATCGCGCCGACCCTGCTCGAGGCGGCGGGGGTGGAGATCTGGCCGGGCATGCAGGGCCGGTCGCTCTGGCCG 1146
                **.**.*****.********.*****.********.******..***.******************************.*.**.***********************.************
                **.**.*****.********.*****.********.******..***.******************************

SUL_Sh          CTGCTGTCGGGCACCGAGACGCAAACCGGCGTCGCGGATGTCTACTGCGAATATTACAACGCCATGCCCTGGCATAAAGAGCCGCATCTGCCATTCGCGACCATGCTGCGCACCGGGACG 1320
SUL_Dev         CTACTCACCGGCCAGGAGACGCAGACCGGGGTCGAGGACGTCTATTGTGAATACTACAACGCCATGCCCTGGCACCGCGAACCGCACCTGCCGTTTGCCACCATGCTGCGCACCGAGACG 1266
                **.**..*.***...********.*****.****.***.*****.**.*****.********************....**.*****.*****.**.**.****************.****

SUL_Sh          ATGAAGCTGGTGGTTAGCCACGGCGACCGGCGCGGTGAGCTTTACGATCTGAATGCAGACCCCGACGAGACCACAAACCTGTGGGACGATCCAAACCGCCTGGCGGAGAAATGCGACCTG 1440
SUL_Dev         ATGAAGCTCGTGGTGAGCCATGGCGACCGGCGCGGCGAGCTCTATGACCTCTTGGCGGACCCCGGAGAAACCACCAATCTGTGGGACGACCCGGATCATCTGGCGGAAAAGGCAGAACTC 1386
                ********.*****.*****.**************.*****.**.**.**....**.*******..**.*****.**.***********.**..*.*..********.**....**.**.

SUL_Sh          TTGCAGCGTCTTTGCGACCGGATGGCCTTCACCGTCGACCCGCTGCCGCTGCGCCGCGCCCCCTGGTAA    1509
SUL_Dev         CTGCAGCGCCTCTGTGACCGGATGGCCTTTACCGTCGACCCGCTGCCGCTGCGCCGCGCCCCCTGGTAG    1455
                .*******.**.**.**************.**************************************.
```

CLUSTAL Omega (1.2.4) sequence alignment of the complete *Shinella* acesulfame sulfatase (SUL_Sh) and *Devosia oryzisoli* PTR5 sulfatase WP_191775306.1 (SUL_Dev) genes. Symbols: * identical; **.** mismatch (highlighted in red); sub-alignment with 38/38 bp identity (100%), with 40/40 bp identity (100%), with 131/145 bp identity (90.3%) and with 130/144 bp identity (90.3%) are shown.

Text S8. Sulfatase-maturating enzymes in the *Shinella* genomes

The SHIWSC3_PJ0001 sulfatase of strain WSC3-e and related enzymes belong to the family of formylglycine-dependent sulfatases. These enzymes need to be activated by formylglycine-generating enzymes (FGEs) that catalyze the posttranslational conversion of a reactive site serine or cysteine residue of the sulfatase into formylglycine. In bacteria, FGEs are either oxygenase-like enzymes that are dependent on molecular oxygen (aerobic sulfatase maturation) or employ a radical *S*-adenosylmethionine mechanism (anaerobic sulfatase maturation).

Genes encoding aerobic sulfatase maturation

According to the genome annotation, two genes encoding aerobic sulfatase-maturating enzymes are present in *Shinella* sp. WSC3-e. The first FGE gene, SHIWSC3_1933, is located on the chromosome (OZ000530). The second one, SHIWSC3_PF0003, is located on plasmid PF (OZ000536). In the proteome of acesulfame-grown *Shinella* sp. WSC3-e, only the SHIWSC3_PF0003 gene product was detected (with a relative abundance of 0.001%, Table S7), indicating that this oxygen-dependent FGE is responsible for the posttranslational modification of the SHIWSC3_PJ0001 sulfatase. An identical copy of the SHIWSC3_PF0003 gene can be found in the other acesulfame-degrading *Shinella* strains, e.g., encoding the FGE MDC7259753.1 in strain YE25 and encoding the FGE MDC7267076.1 in HY16.

Genes encoding anaerobic sulfatase maturation

The genome annotations did not present any gene encoding an anaerobic sulfatase-maturating enzyme. Additionally, a tblastn search with two reference sequences of anaerobic sulfatase-maturating enzymes (cysteine-type anaerobic sulfatase-maturating enzyme from *Clostridium perfringens*, UniProt ID: Q0TTH1,[13] serine-type anaerobic sulfatase-maturating enzyme from *Klebsiella pneumoniae*, UniProt ID: Q9X758[14]) as query against the *Shinella* sp. WSC3-e genome did not detect any oxygen-independent FGE employing the S-adenosylmethionine mechanism. In fact, genes encoding these enzymes are not present in any published genome of *Shinella* isolates (NCBI tblastn search, July 2024), including the denitrifying strains YE25 and HY16.

Supplementary Figures

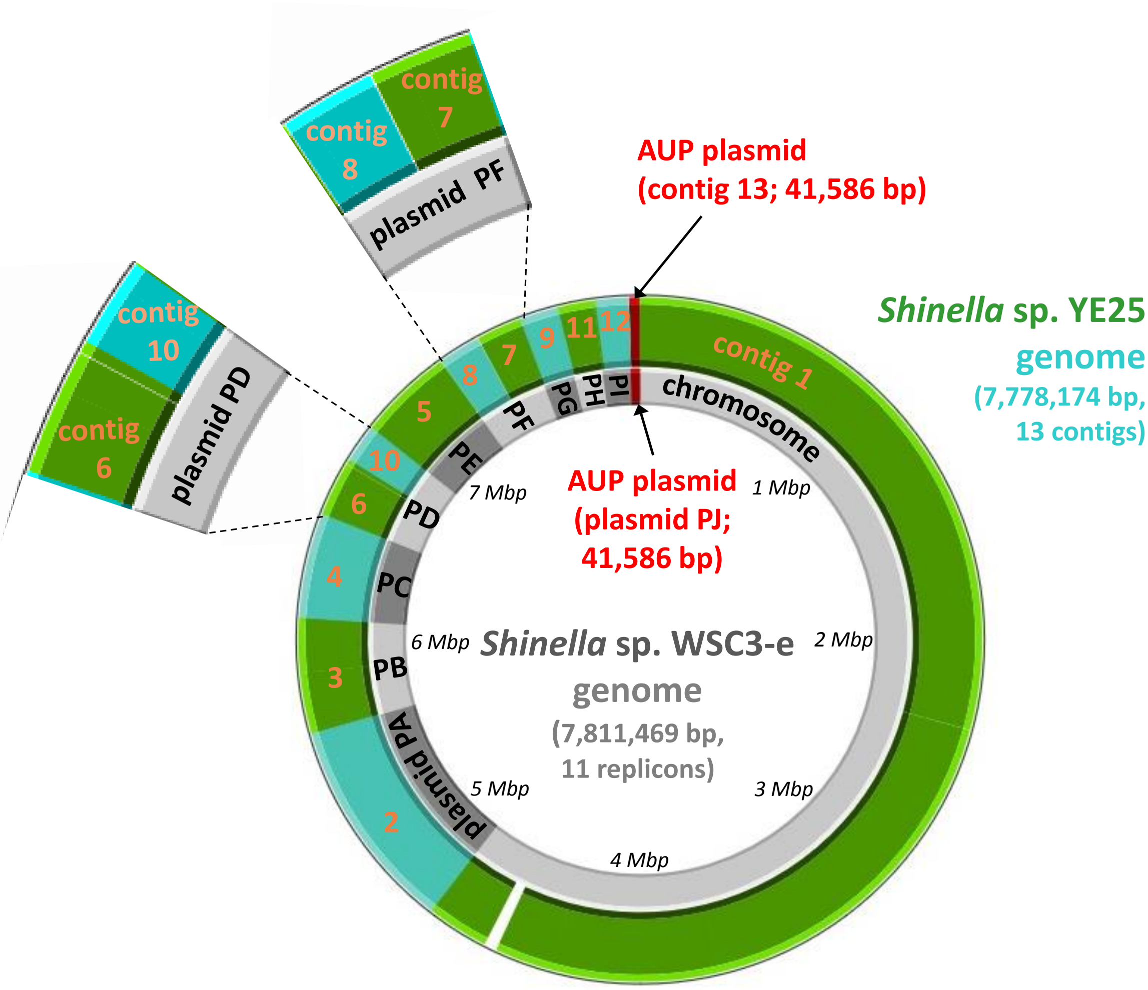

Figure S1. Alignment of the *Shinella* sp. YE25 genome (green and turquoise) against the *Shinella* sp. WSC3-e genome (gray).

Respective contig numbers/replicon names are indicated (orange and black). Both strains share almost identical genome sequence (≥99%) and replicon organization, suggesting the presence of a large chromosome (about 4.7 Mbp) and several plasmids of various size. The smallest plasmid, for example, is the AUP plasmid (41,586 bp, highlighted in red) which is 100% identical in both strains. In contrast, a section (ca. 30 kbp) close to the end of the WSC3-e chromosome is completely missing in strain YE25. In addition, plasmids PD and PF of strain WSC3-e can be derived from smaller YE25 plasmids. In each case, two plasmids were rearranged to a larger one (see enlargement). The figure was generated with Proksee.

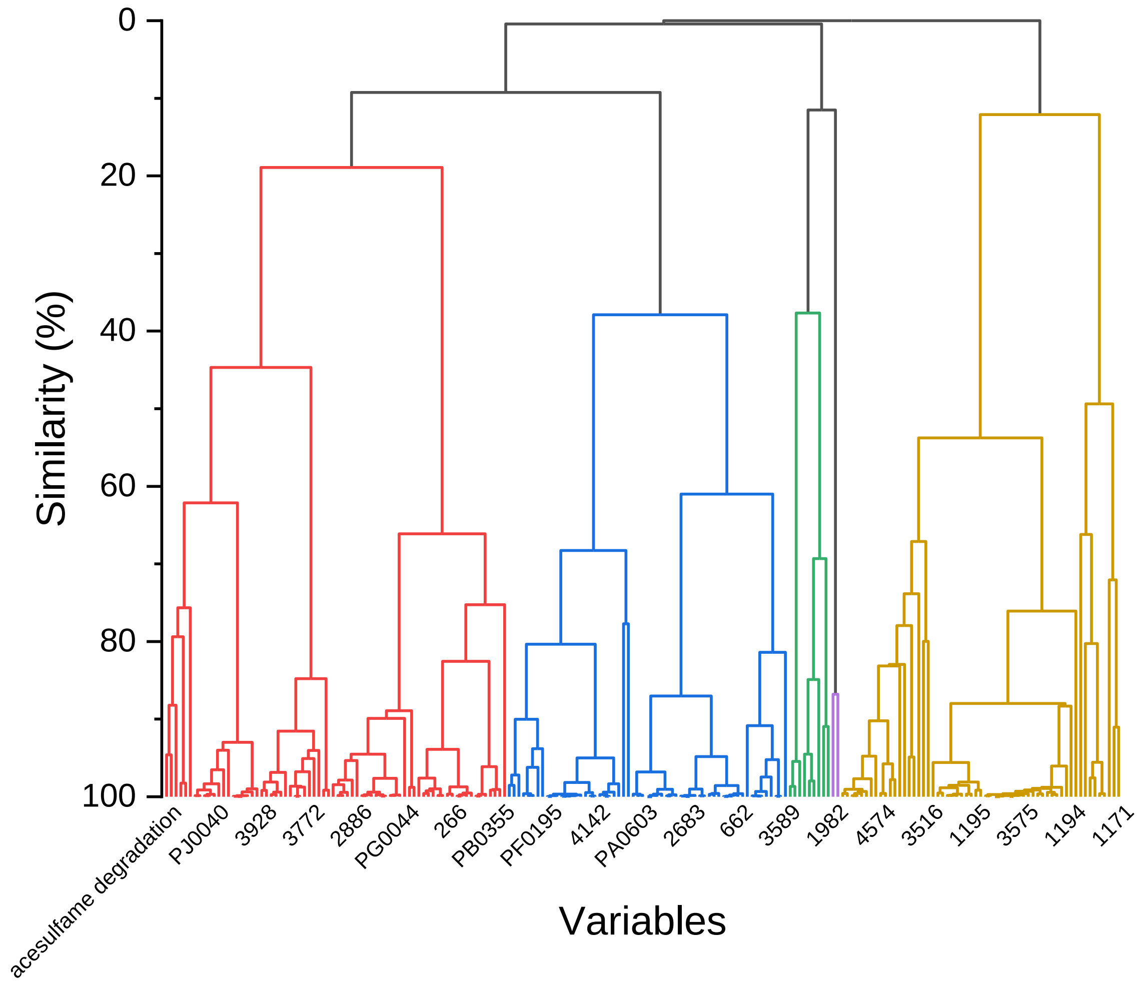

Figure S2. Dendrogram of hierarchical cluster analysis for acesulfame degradation and abundance distribution of 200 most abundant *Shinella* sp. WSC3-e proteins after summing up the proteins in 30 SEC fractions.

Protein names indicated refer to locus tags (prefix SHIWSC3_). Not all protein names are shown due to space limitation. The protein names shown were automatically decided by the Origin software.

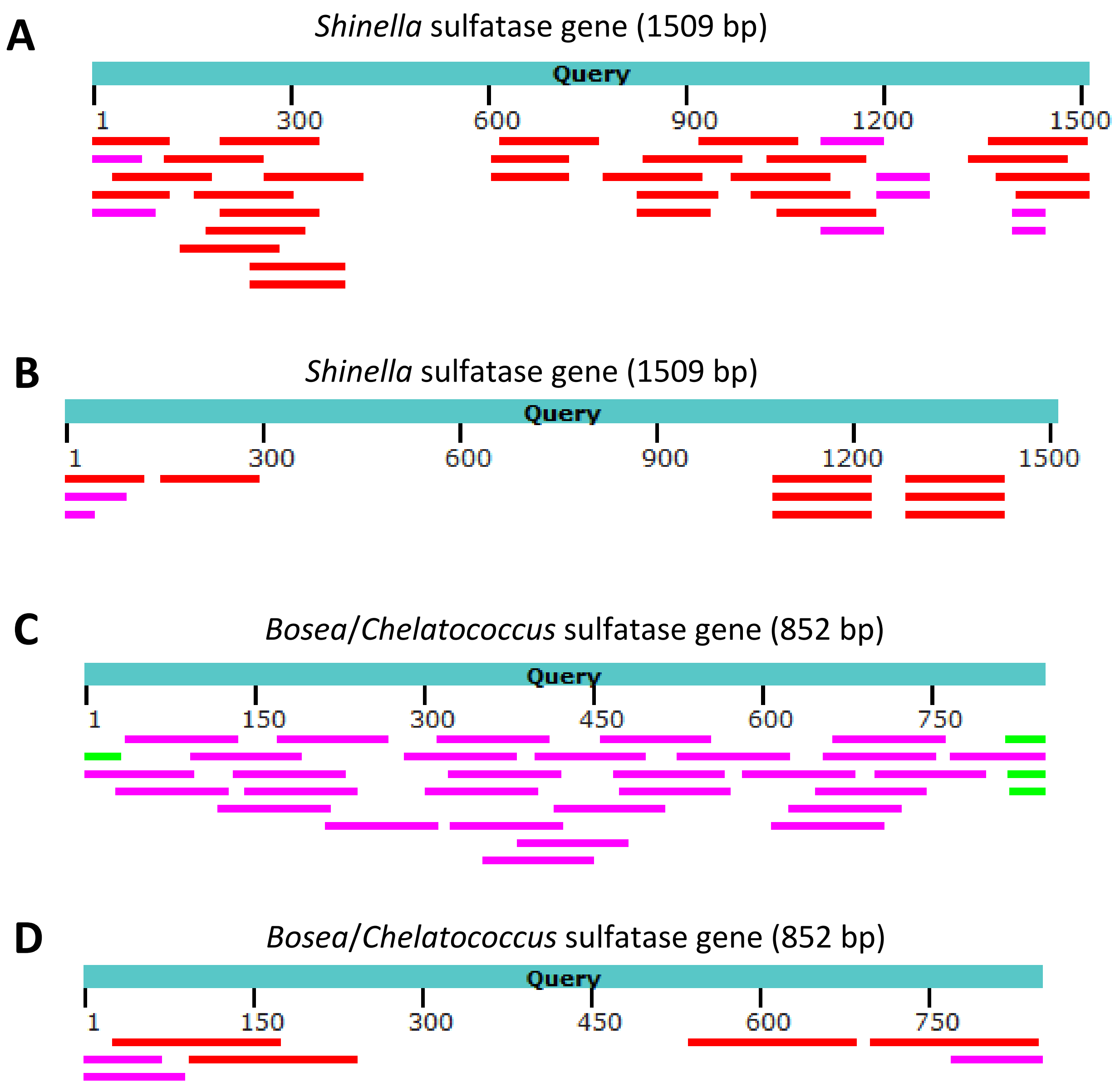

Figure S3. Examples for blastn results with acesulfame sulfatase genes from acesulfame-degrading *Bosea*/*Chelatococcus* and *Shinella* strains as query against datasets from the SRA database.

For the alignments, only sequences showing ≥97% identity to the query sequence were considered. Color code as used by the NCBI blast graphic summary showing alignment scores (≥200, red; 80 – 200, magenta; 50 – 80, green). (A) Search with SHIWSC3_PJ0001 against SRA files from project PRJNA807808 (150-bp reads; pilot-scale microalgae-bacteria wastewater treatment system, Querétaro, Mexico, sampled in 2020). (B) Search with SHIWSC3_PJ0001 against SRA files from project PRJNA988425 (150-bp reads; WWTP in Xinjiang, China, sampled in 2021). (C) Search with BOSEA1005_40015 against SRA files from project PRJNA967004 (100-bp reads; WWTP in Galway, Ireland, sampled in 2020 and 2021). (D) Search with BOSEA1005_40015 against SRA files from project PRJNA952735 (150-bp reads; WWTP in Regina, Canada, sampled in 2017).

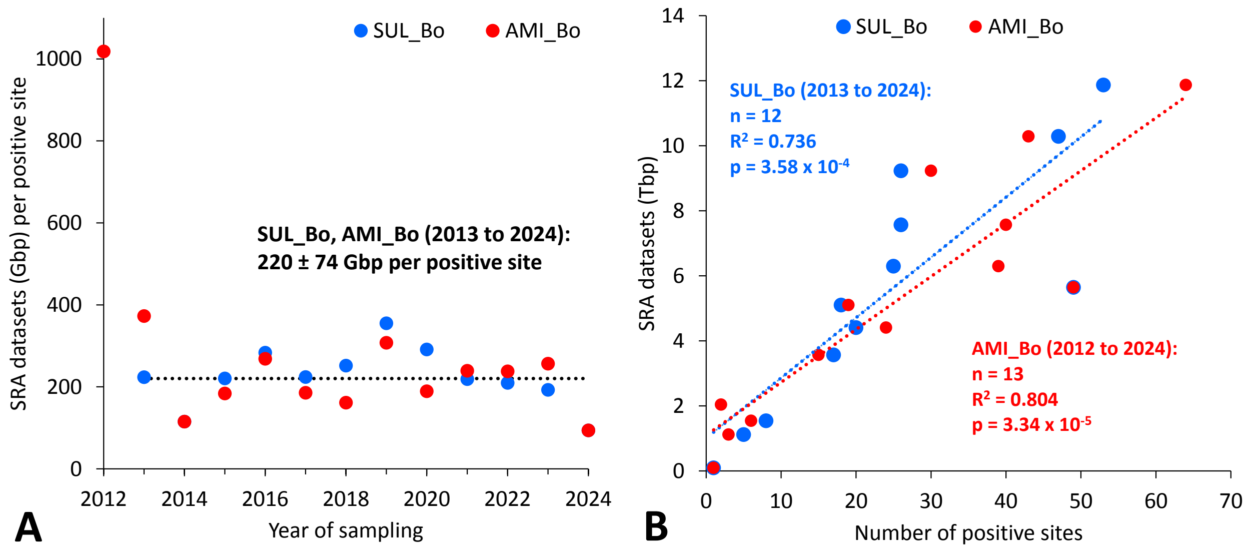

Figure S4. Historic occurrence of *Bosea*/*Chelatococcus* acesulfame sulfatase (SUL_Bo) and ANSA amidase (AMI_Bo) gene signatures in shotgun metagenome and metatranscriptome SRA datasets related to wastewater, WWTP or receiving waters across all populated continents.

(A) Ratio of SRA datasets searched and the number of sites with detection of SUL_Bo and AMI_Bo gene signatures for the sampling years 2012 to 2024. The mean value and SD for the detection of both genes in 2013 to 2024 is indicated (dotted line). (B) Correlation between number of sites with detection of the gene signatures and size of SRA datasets searched for the sampling years 2012 to 2024 (AMI_Bo) and 2013 to 2024 (SUL_Bo). For both linear regression analyses (dotted lines), R^2 and p-values are given. The correlation analysis was done using the linear regression and FDIST functions in Excel (Microsoft Corporation, Redmond, Washington, USA).

References

(1) Kolmogorov, M.; Bickhart, D. M.; Behsaz, B.; Gurevich, A.; Rayko, M.; Shin, S. B.; Kuhn, K.; Yuan, J.; Polevikov, E.; Smith, T. P. L.; Pevzner, P. A., MetaFlye: scalable long-read metagenome assembly using repeat graphs. *Nat. Methods.* **2020,** *17* (11), 1103-1110.

(2) Walker, B. J.; Abeel, T.; Shea, T.; Priest, M.; Abouelliel, A.; Sakthikumar, S.; Cuomo, C. A.; Zeng, Q.; Wortman, J.; Young, S. K.; Earl, A. M., Pilon: an integrated tool for comprehensive microbial variant detection and genome assembly improvement. *PLOS ONE.* **2014,** *9* (11), e112963.

(3) Bonatelli, M. L.; Rohwerder, T.; Popp, D.; Liu, Y.; Akay, C.; Schultz, C.; Liao, K.-P.; Ding, C.; Reemtsma, T.; Adrian, L.; Kleinsteuber, S., Recently evolved combination of unique sulfatase and amidase genes enables bacterial degradation of the wastewater micropollutant acesulfame worldwide. *Front. Microbiol.* **2023,** *14*.

(4) Schopper, S.; Kahraman, A.; Leuenberger, P.; Feng, Y.; Piazza, I.; Muller, O.; Boersema, P. J.; Picotti, P., Measuring protein structural changes on a proteome-wide scale using limited proteolysis-coupled mass spectrometry. *Nat Protoc* **2017,** *12* (11), 2391-2410.

(5) Ding, C.; Adrian, L., Comparative genomics in "*Candidatus* Kuenenia stuttgartiensis" reveal high genomic plasticity in the overall genome structure, CRISPR loci and surface proteins. *BMC Genomics.* **2020,** *21* (1), 851.

(6) Loman, N. J.; Misra, R. V.; Dallman, T. J.; Constantinidou, C.; Gharbia, S. E.; Wain, J.; Pallen, M. J., Performance comparison of benchtop high-throughput sequencing platforms. *Nat. Biotechnol.* **2012,** *30* (5), 434-9.

(7) Meacham, F.; Boffelli, D.; Dhahbi, J.; Martin, D. I. K.; Singer, M.; Pachter, L., Identification and correction of systematic error in high-throughput sequence data. *BMC Bioinformatics* **2011,** *12* (1), 451.

(8) van Dijk, E. L.; Auger, H.; Jaszczyszyn, Y.; Thermes, C., Ten years of next-generation sequencing technology. *Trends Genet* **2014,** *30* (9), 418-26.

(9) Gilles, A.; Meglécz, E.; Pech, N.; Ferreira, S.; Malausa, T.; Martin, J.-F., Accuracy and quality assessment of 454 GS-FLX Titanium pyrosequencing. *BMC Genomics.* **2011,** *12* (1), 245.

(10) Mascher, M.; Wu, S.; Amand, P. S.; Stein, N.; Poland, J., Application of Genotyping-by-Sequencing on Semiconductor Sequencing Platforms: A Comparison of Genetic and Reference-Based Marker Ordering in Barley. *PLOS ONE.* **2013,** *8* (10), e76925.

(11) Zhang, H.; Jain, C.; Aluru, S., A comprehensive evaluation of long read error correction methods. *BMC Genomics.* **2020,** *21* (6), 889.

(12) Blin, K. *ncbi-genome-download*, **2023-07-28**.

(13) Nguyen, T. D.; Liu, Y.; Saha, S.; Leung, K. C. F.; Stoddart, J. F.; Zink, J. I., Design and Optimization of Molecular Nanovalves Based on Redox-Switchable Bistable Rotaxanes. *J. Am. Chem. Soc.* **2007,** *129* (3), 626-634.

(14) Grove, T. L.; Lee, K. H.; St Clair, J.; Krebs, C.; Booker, S. J., In vitro characterization of AtsB, a radical SAM formylglycine-generating enzyme that contains three [4Fe-4S] clusters. *Biochem.* **2008,** *47* (28), 7523-38.
